# Explosive cell lysis as a mechanism for the biogenesis of bacterial membrane vesicles and biofilms

**DOI:** 10.1038/ncomms11220

**Published:** 2016-04-14

**Authors:** Lynne Turnbull, Masanori Toyofuku, Amelia L. Hynen, Masaharu Kurosawa, Gabriella Pessi, Nicola K. Petty, Sarah R. Osvath, Gerardo Cárcamo-Oyarce, Erin S. Gloag, Raz Shimoni, Ulrich Omasits, Satoshi Ito, Xinhui Yap, Leigh G. Monahan, Rosalia Cavaliere, Christian H. Ahrens, Ian G. Charles, Nobuhiko Nomura, Leo Eberl, Cynthia B. Whitchurch

**Affiliations:** 1 The ithree institute, University of Technology Sydney, Ultimo, New South Wales 2007, Australia; 2 Department of Life and Environmental Sciences, University of Tsukuba, Tsukuba, Ibaraki 305-8572, Japan; 3 Department of Plant and Microbial Biology, University of Zurich, Zürich 8008, Switzerland; 4 Department of Biology, Institute of Molecular Systems Biology, ETH Zurich, Zürich 8093, Switzerland; 5 Agroscope, Institute for Plant Production Sciences, Research Group Molecular Diagnostics, Genomics and Bioinformatics, & Swiss Institute of Bioinformatics (SIB), Wädenswil 8820, Switzerland

## Abstract

Many bacteria produce extracellular and surface-associated components such as membrane vesicles (MVs), extracellular DNA and moonlighting cytosolic proteins for which the biogenesis and export pathways are not fully understood. Here we show that the explosive cell lysis of a sub-population of cells accounts for the liberation of cytosolic content in *Pseudomonas aeruginosa* biofilms. Super-resolution microscopy reveals that explosive cell lysis also produces shattered membrane fragments that rapidly form MVs. A prophage endolysin encoded within the R- and F-pyocin gene cluster is essential for explosive cell lysis. Endolysin-deficient mutants are defective in MV production and biofilm development, consistent with a crucial role in the biogenesis of MVs and liberation of extracellular DNA and other biofilm matrix components. Our findings reveal that explosive cell lysis, mediated through the activity of a cryptic prophage endolysin, acts as a mechanism for the production of bacterial MVs.

## Abstract (web summary)

Many bacteria release DNA and membrane vesicles through unclear mechanisms. Here, the authors show that a prophage endolysin is involved in the explosive lysis of a sub-population of cells in *Pseudomonas aeruginosa*, releasing cytoplasmic content and membrane fragments that rapidly form membrane vesicles.

## Graphical abstract

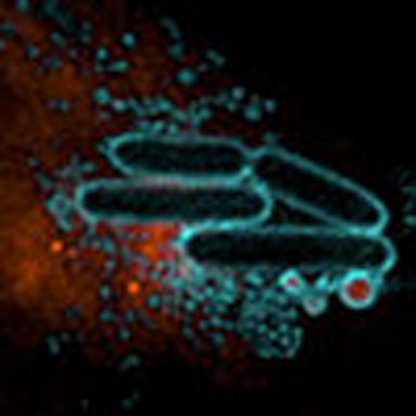

Both Gram-negative and Gram-positive bacteria produce membrane vesicles (MVs) that have been shown to contribute to diverse biological processes, including biofilm development, virulence, quorum sensing, phage decoy and horizontal gene transfer. MVs are bi-layered spheres which, at least in Gram-negative bacteria, are thought to be produced through blebbing of the outer membrane and hence are often referred to as outer-membrane vesicles^1^,^2^. MVs derived from planktonic cultures have been the most extensively studied and have been found to be comprised of outer-membrane proteins, lipopolysaccharide and to encapsulate periplasmic components including peptidoglycan and virulence factors^1^,^2^. Interestingly, MVs also contain numerous inner membrane and cytoplasmic proteins, as well as DNA and RNA^1^,^2^. MVs are also present in biofilms where they interact with extracellular DNA (eDNA) in the biofilm matrix to enhance structural integrity and to serve as decoys to protect biofilm cells from antibiotics^1^,^2^,^3^,^4^.

The matrix of bacterial biofilms is a complex mixture of exopolysaccharides, nucleic acids, proteins and MVs that serve as public goods for the biofilm community by providing important functions including self-organization, surface adhesion, intercellular connectivity, structural integrity, cell–cell communication, virulence, nutrient acquisition and antibiotic resistance^1^,^2^,^3^,^5^,^6^,^7^,^8^,^9^. A number of cytosolic proteins have also been shown to have moonlighting roles in biofilm formation or virulence when released from the cytosol of the cell^10^,^11^. It is currently unclear how many of these biofilm matrix components and moonlighting proteins are liberated into the extracellular milieu or transported to the cell surface.

It is now evident that eDNA is a common feature of biofilms formed by many bacterial species and its production has been attributed to autolysis, phage-mediated cell lysis or active secretion systems^8^,^12^. We have shown previously that eDNA is essential for biofilm formation by the Gram-negative bacterium *Pseudomonas aeruginosa* (*P. aeruginosa*)^9^ and that eDNA facilitates the self-organization of *P. aeruginosa* biofilms as they actively migrate across surfaces via twitching motility^7^. In this study, we show that eDNA is produced by *P. aeruginosa* through explosive cell lysis events mediated by a cryptic prophage endolysin encoded in the R- and F-pyocin gene cluster. Using live-cell super-resolution imaging we show that these explosive cell lysis events also produce MVs through vesicularization of shattered membrane fragments.

## Results

### Explosive cell lysis occurs in interstitial biofilms

**Figure 1:**
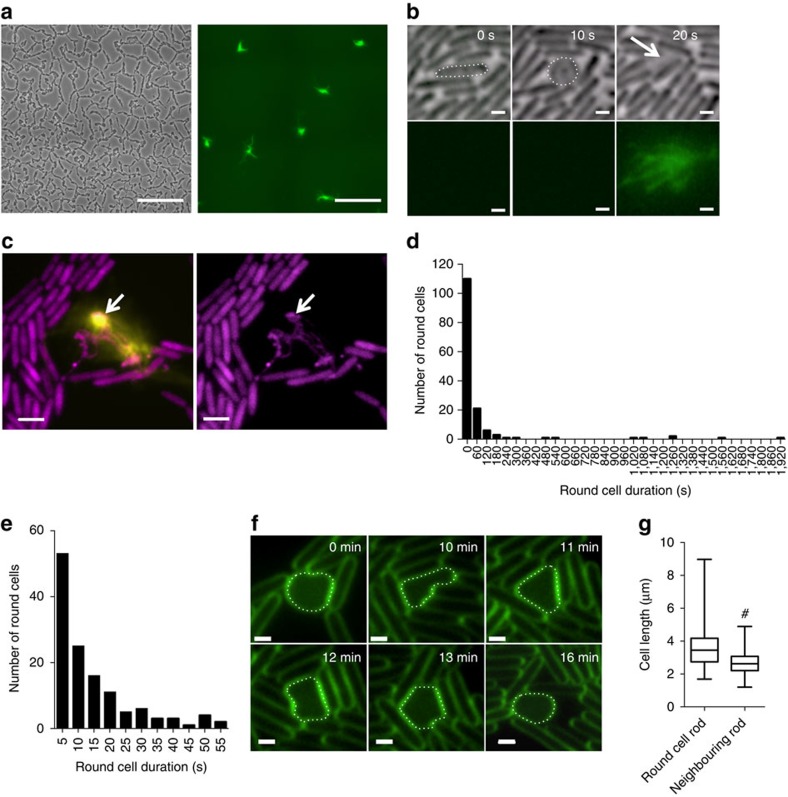
Explosive cell lysis occurs in *P. aeruginosa* interstitial biofilms. (**a**) Phase-contrast (left) and TOTO-1-stained eDNA (green, right); scale bar, 50 μm. (**b**) Time series of a rod-to-round cell transition (dotted white line, upper panels) and subsequent lysis releasing eDNA stained by TOTO-1 (green, lower panels). Time in seconds (top right); scale bar, 1 μm. (**c**) *P. aeruginosa* PAK-expressing cytoplasmic CFP (magenta) cultured in the presence of the eDNA stain TOTO-1 (yellow) showing that sites of eDNA release (arrow, left panel) contain extracellular CFP (arrow, right panel); scale bar, 2 μm. (**d**) Frequency distribution of survival times in seconds (s) of round cells from formation to explosion (*n*=150, bin size 60s). Another 12 round cells were observed that had either formed within a time-series or were present at the start of a time-series and which did not explode by the end of the time-series. Survival times of these cells were at least 10–45 min including one that we tracked for several hours. (**e**) Frequency distribution of round cell survival for those cells surviving <60 s (*n*=129, bin size 5s). (**f**) A round cell (dotted white line) cultured in the presence of FM-143FX (green), tracked over 20 min. Time in min (top right); scale bar, 1 μm. These round cells with long survival times had malleable cell walls and were able to withstand being pushed out of shape by surrounding cells and rapidly re-formed the default round shape when the neighbouring cells moved away. See Supplementary Movie 2. (**g**) Cell lengths of round cells that became rods (round cell rod; *n*=120) and neighbouring rod cells (*n*=750). Box is 25th–75th percentiles, line in box is median, whisker limits are minimum and maximum values; #*P*<0.0001, unpaired *t*-test with Welch's correction.

We have observed that in actively expanding interstitial biofilms of *P. aeruginosa* strain K (PAK), eDNA is distributed as numerous bright foci throughout the active migration zone of the biofilm^7^ (Fig. 1a). This pattern suggests that eDNA is produced by discrete cells rather than generalized secretion by the whole population. To determine the mechanism by which eDNA is produced in interstitial biofilms we followed 150 eDNA release events and found that all were associated with the rapid transition of rod-shaped cells to round cells that subsequently exploded resulting in the annihilation of the cell and release of eDNA into the environment (Fig. 1b; Supplementary Movie 1).

As explosive cell lysis is an effective mechanism for the release of eDNA, we considered that this process would also liberate cytoplasmic proteins into the extracellular milieu. Indeed, examination of biofilms of *P. aeruginosa* PAK expressing cytoplasmic cyan fluorescent protein (CFP) revealed that areas of extracellular CFP are co-located with eDNA (Fig. 1c). These observations suggest that explosive cell lysis is also an effective mechanism for the liberation of cytoplasmic proteins.

The rate of transition from the rod to round cell morphotype is extremely rapid occurring in <5–10 s (Fig. 1b; Supplementary Movie 1). We analysed the survival times of 150 *P. aeruginosa* PAK round cells and found that 86% of these survived for <60 s with 35% surviving for <5 s, although some round cells had much longer survival times (Fig. 1d–f; Supplementary Movie 2). The bacterial cell wall is the primary stress-bearing structure that dictates and maintains cell shape and protects the cytoplasmic membrane against turgor and lysis^13^. Our observations suggest that the transition from rod to round cell morphotype involves the rapid loss of structural integrity of the cell wall peptidoglycan, which in most instances results in immediate explosive cell lysis. We investigated the viability of round cells by culturing *P. aeruginosa* interstitial biofilms in the presence of the live-cell impermeant nucleic acid stain ethidium homodimer-2 (EtHD-2) which is a sensitive marker of dead microbial cells as it only enters cells with damaged membranes^14^. We found that round cells excluded EthHD-2 indicating that they have intact membranes and are likely to be viable until the explosion event (Supplementary Movie 3). Our analyses also revealed that many of the rod cells that subsequently became round cells were frequently longer than the surrounding cells, including some extremely long cells that were up to 3–4 times longer than the average size of the neighbouring rod cells (Fig. 1g). Of 156 rod cells that became round cells, 33 were clearly undergoing cell division. These observations suggest that some round cells originated from rod cells that were either undergoing cell division or were blocked in cell division.

To determine if explosive cell lysis was a conserved phenomenon in *P. aeruginosa* strains, we cultured interstitial biofilms of the common laboratory strains PAK, PAO1, PA103, PA14, ATCC27853, five CF clinical isolates and two non-CF clinical isolates, and quantified the frequency of round cells and sites of eDNA release as a marker for explosive cell lysis. To quantify the frequency of round cells in *P. aeruginosa* interstitial biofilms, randomly selected fields of view of the interstitial biofilm monolayer were imaged with phase contrast microscopy and analysed via computer vision to identify cells and categorize their morphotypes as rod or round cells. Round cells were observed in all strains, although the frequency of round cells varied from about 1 per 3,000 to 1 per 100,000 rod cells in different strains (Supplementary Fig. 1a). Similar frequencies of punctate eDNA sites were observed in the interstitial biofilms of all strains (Supplementary Fig. 1b). These observations suggest that while eDNA release through explosive cell lysis occurs in many *P. aeruginosa* strains, the survival times of round cells appear to vary between strains that is reflected in the number of round cells visible in the population at any instant in time.

### Explosive cell lysis is induced by stress

**Figure 2:**
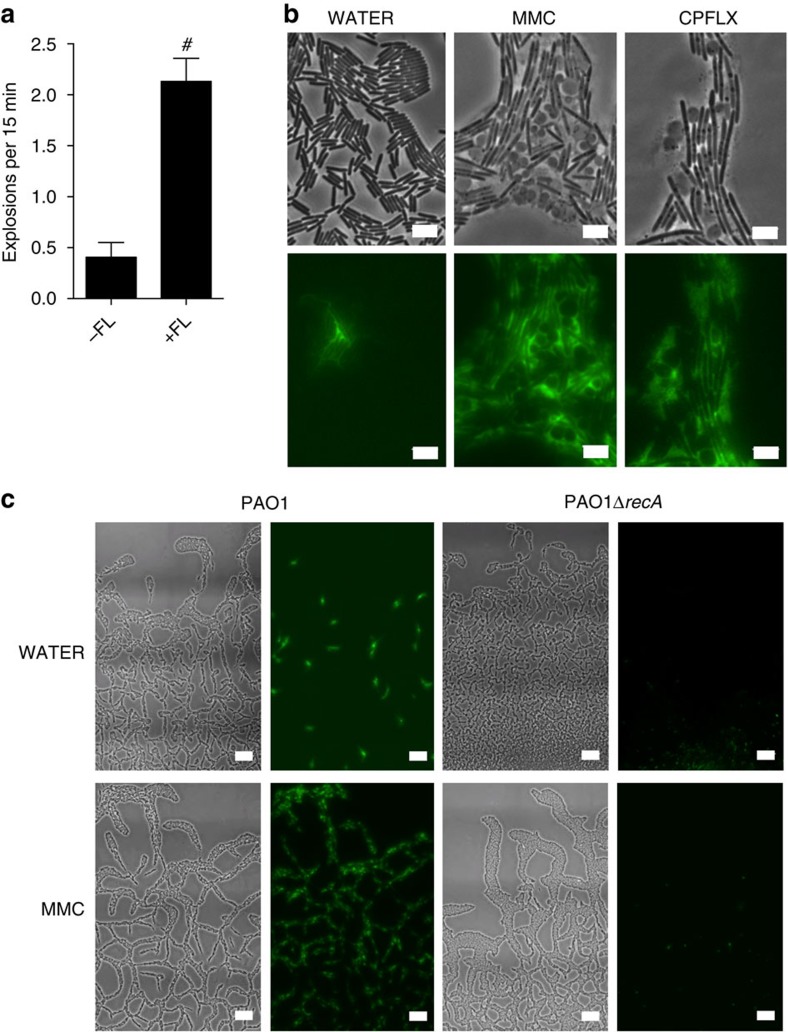
Stress induces explosive cell lysis. (**a**) Frequency of explosive cell lysis events in the absence (−FL) or presence (+FL) of fluorescence imaging, mean±s.e.m., #*P*<0.0001, Unpaired *t*-test with Welch's correction. (**b**) Phase-contrast (top) and TOTO-1-stained eDNA (green, bottom) of *P. aeruginosa* PAO1 interstitial biofilms cultured in the presence of filter discs saturated in water, MMC or CPFLX; scale bar, 5 μm. (**c**) Phase-contrast (left) and TOTO-1-stained eDNA (green, right) of *P. aeruginosa* PAO1 and PAO1Δ*recA* interstitial biofilms cultured in the presence of filter discs saturated in water, or MMC; scale bar, 20 μm.

Interestingly, we noted that fluorescence imaging in the presence of the eDNA stain TOTO-1 to detect eDNA release events yielded more frequent explosive cell lysis events than when fluorescence imaging was not involved (Fig. 2a) although the process appeared identical (Supplementary Movie 4). This suggests that phototoxicity due to exposure to high-intensity excitation light stimulated explosive cell lysis. To determine if other sources of exogenous stress induce explosive cell lysis, we examined the effect of exposing *P. aeruginosa* interstitial biofilms to antibiotic stress (ciprofloxacin; CPFLX) or genotoxic stress (mitomycin C; MMC). We found that as the biofilm approached CPFLX or MMC gradients, round cells were induced and large quantities of eDNA released through explosive cell lysis (Fig. 1b). To determine if induction of explosive cell lysis by these exogenous stresses is mediated by the RecA-mediated SOS stress response regulon of *P. aeruginosa*, we examined the response of a PAO1Δ*recA* mutant and found no evidence of explosive cell lysis in this strain under either inducing or non-inducing conditions (Fig. 2c).

### Lys endolysin mediates explosive cell lysis

The cell rounding and explosive cell lysis events that we have described here appear very similar to the release of lytic bacteriophages from host cells^15^. Moreover, DNA-damaging agents such as MMC or CPFLX are known to induce prophages in a RecA-dependent manner^16^. The *P. aeruginosa* PAO1 genome contains a cluster of genes that encodes the R- and F-type pyocins that are cryptic prophages related to the lytic bacteriophages P2 and lambda, respectively^17^. The R- and F-pyocin gene cluster encodes the only putative bacteriophage-like endolysin that we could identify on the PAO1 genome (PA0629, previously termed *lys*^17^). Interestingly, Nakayama *et al*.^17^ have shown that overexpression of PA0629 in *P. aeruginosa* causes cell lysis and have proposed a model in which the holin Hol (PA0614) disrupts the inner membrane thereby allowing the endolysin Lys (PA0629) to translocate to the periplasm where it degrades the peptidoglycan to release the pyocins. As is common with many prophages, the production of the R- and F-pyocins is induced through the RecA-mediated SOS response of *P. aeruginosa*^12^,^17^,^18^,^19^,^20^,^21^.

**Figure 3:**
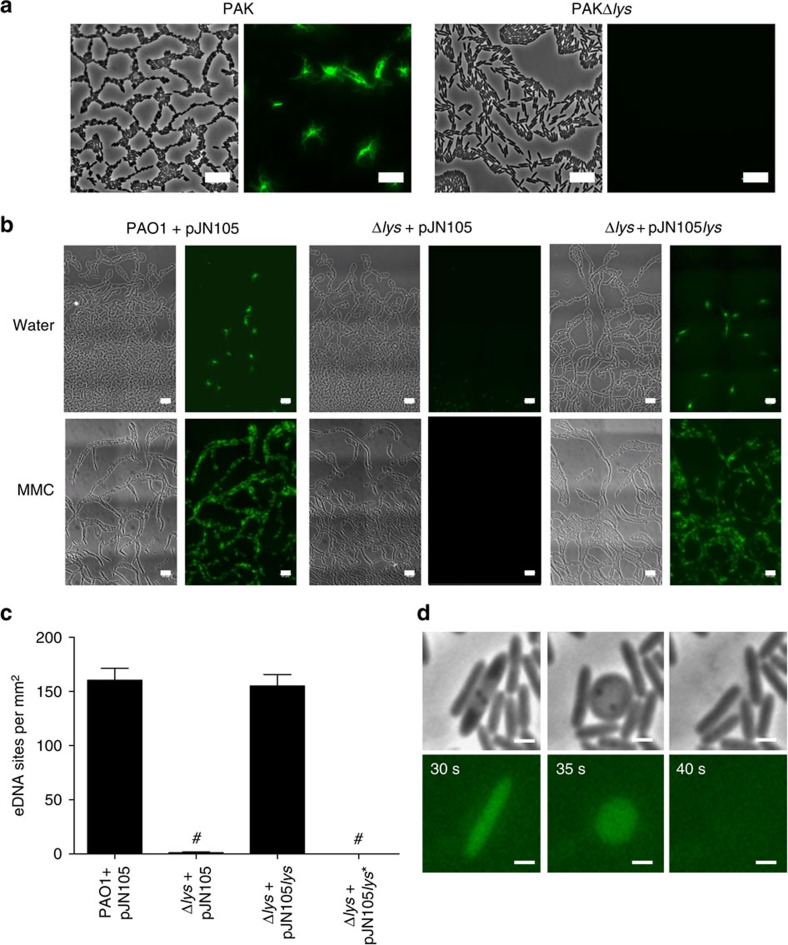
Pyocin endolysin Lys is required for eDNA release in interstitial biofilms under inducing and non-inducing conditions. (**a**) and (**b**) Phase-contrast (left) and TOTO-1-stained eDNA (green, right) of interstitial biofilms of (**a**) PAK and PAKΔ*lys* and (**b**) PAO1 and PAO1Δ*lys* containing either pJN105 (vector control) or pJN105*lys* cultured in the presence of filter discs saturated in water or MMC; scale bar, 10 μm. (**c**) Lys catalytic activity is required for eDNA release in *P. aeruginosa* PAO1 interstitial biofilms; *n*=30; mean±s.e.m. #*P*<0.0001, unpaired *t*-test with Welch's correction. (**d**) Time series of cells in an interstitial biofilm of *P. aeruginosa* PAO1 (phase-contrast, upper panels) containing the *P*_*hol*_-*eGFP* transcriptional fusion on plasmid pM0614-G (green, lower panels). Time in seconds; scale bar, 1 μm.

We therefore explored the possibility that the putative endolysin Lys may be responsible for eDNA release through explosive cell lysis in *P. aeruginosa* biofilms. We found that PAO1Δ*lys* and PAKΔ*lys* mutants were significantly abrogated in the explosive cell lysis-mediated release of eDNA in interstitial biofilms under both inducing and non-inducing conditions (Fig. 3a,b). Explosive cell lysis was restored with wild-type *lys* provided *in trans* but not by the mutant allele lys* that encodes an E51V substitution in the putative active site (Fig. 3b,c; Supplementary Fig. 2), indicating that the endolytic activity of Lys is critical for explosive cell lysis in these interstitial biofilms. Furthermore, PAKΔ*lys* interstitial biofilms lacked the intricate trail networks that are a characteristic feature of wild-type PAK *P. aeruginosa* interstitial biofilms (Fig. 3a) and appeared morphologically similar to PAK interstitial biofilms cultured in the presence of DNaseI^7^. This indicates that Lys-mediated explosive cell lysis is likely to be the major source of eDNA that is required for self-organization of these interstitial biofilms.

To further examine the correlation between pyocin gene expression and explosive cell lysis, we utilized a *P*_*hol*_-eGFP transcriptional fusion. We followed 74 explosive cell lysis events and found that all of the exploding cells had high levels of eGFP expression compared with the neighbouring rod-shaped cells indicating that the expression of pyocin genes is upregulated in these cells (Fig. 3d; Supplementary Movie 5). We also examined the involvement of genes encoding structural components of the R- and F-pyocins and found that none of the other pyocin gene mutants examined showed any defect in eDNA release in interstitial biofilms (Supplementary Fig. 3). Taken together, these observations indicate that the pyocin endolysin Lys, but not pyocins *per se*, is required for eDNA production via explosive cell lysis in interstitial biofilms of *P. aeruginosa*.

### Lys mediates eDNA release in submerged biofilms

**Figure 4:**
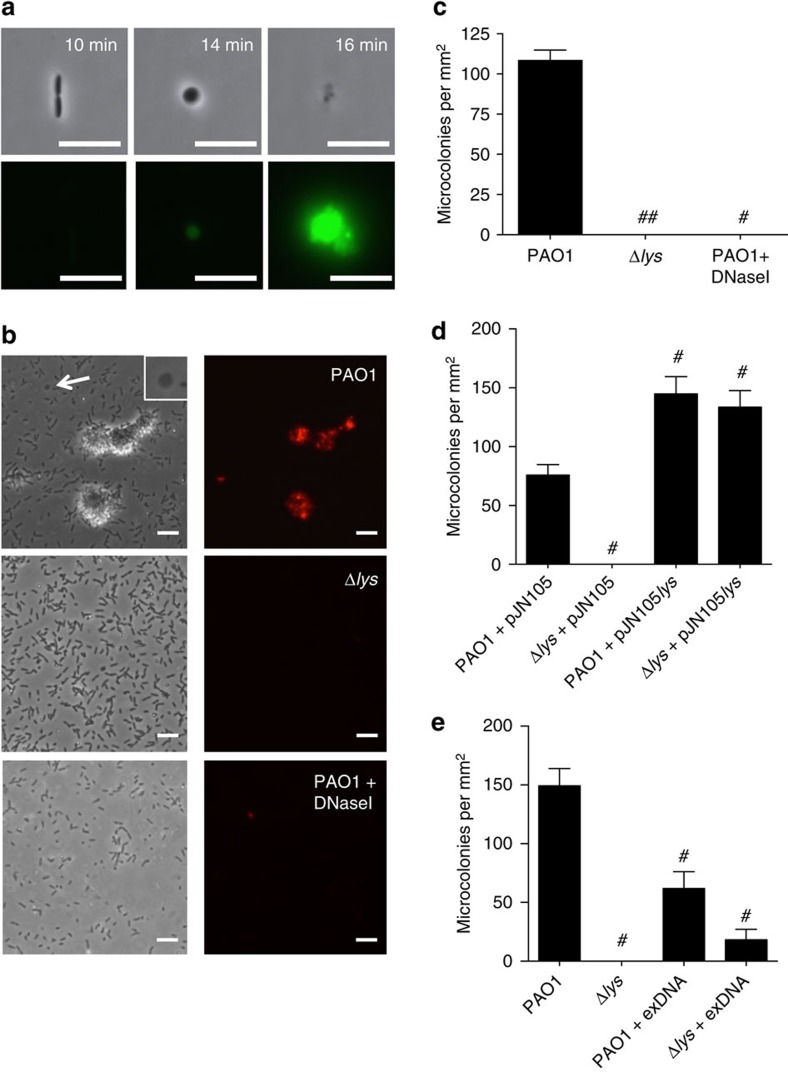
Explosive cell lysis is required for microcolony development in submerged hydrated biofilms. (**a**) Time series of the initial stages of PAO1 biofilm development 1 h after inoculation showing attachment of a rod cell, its transition to round cell morphotype and subsequent explosion releasing eDNA (TOTO-1, green). Time in min (top right); scale bar, 5 μm. (**b**,**c**) Microcolonies in 8-h submerged biofilms of PAO1 (upper), PAO1Δ*lys* (middle), and PAO1 cultured in the presence of DNaseI (lower). (**b**) Representative phase contrast (left) and eDNA (EthHD-2, right) images; scale bar, 10 μm. Inset shows magnified view of round cell at arrow-head (**c**) Microcolonies in 8-h submerged biofilms per mm^2^, *n*=30. Mean±s.e.m. *P<0.0001, unpaired *t*-test with Welch's correction. (**d**) Microcolonies per mm^2^ in 8-h submerged hydrated biofilms of PAO1 and PAO1Δ*lys* carrying either pJN105 or pJN105*lys*, *n*=30. Mean±s.e.m. #*P*<0.0001, unpaired *t*-test with Welch's correction. (**e**) Microcolonies per mm^2^ in 8-h submerged hydrated biofilms of PAO1 and PAO1Δ*lys* cultured in the absence or presence of exogenous DNA (exDNA), *n*=20. Mean±s.e.m. #*P*<0.0001, unpaired *t*-test with Welch's correction.

We have shown previously that eDNA plays an essential role in the early stages of the development of *P. aeruginosa* biofilms that are formed on abiotic surfaces submerged in liquid nutrient media^9^. To determine if explosive cell lysis accounts for eDNA release during the initial stages of the development of submerged biofilms, we performed live-cell imaging of the very early stages of biofilm development and observed the formation and explosion of round cells in both the planktonic phase and at the surface (Fig. 4a; Supplementary Movie 6). In contrast, we were unable to observe any round cells or explosive cell lysis events in PAO1Δ*lys*.

**Figure 5:**
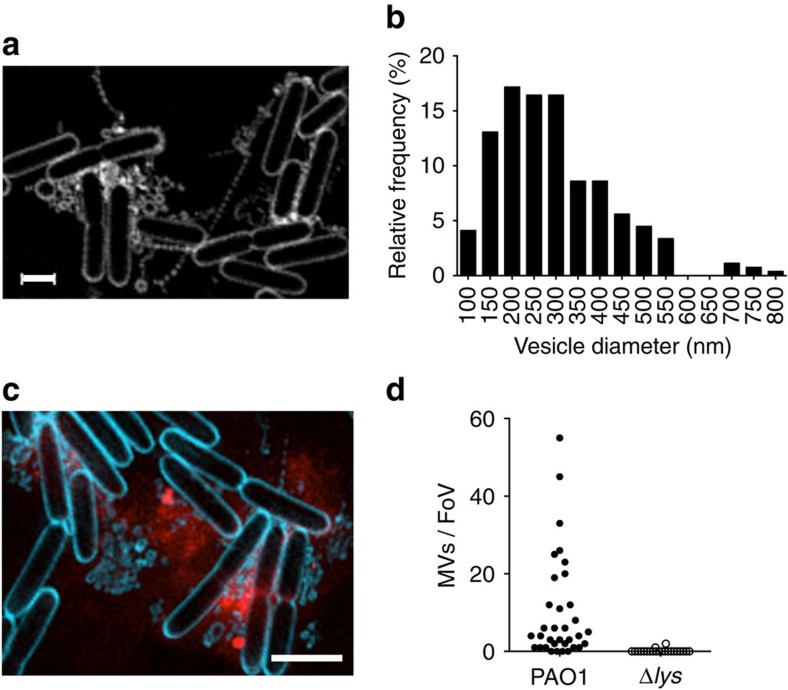
MVs are present within *P. aeruginosa* interstitial biofilms. (**a**) f3D-SIM of PAK biofilms cultured in the presence of FM1-43FX (white), scale bar, 1 μm. (**b**) Frequency distribution of diameters of MVs measured *in situ* in live PAK biofilms (*n*=268, bin size=50 nm). (**c**) f3D-SIM of PAK biofilms cultured in the presence FM1-43FX (blue) and EthHD-2 (red); scale bar, 2 μm. (**d**) Quantification of MVs in random fields of view (40 μm × 40 μm) of PAO1 (*n*=35) and PAO1Δ*lys* (*n*=22) biofilms cultured in the presence of FM1-43FX and imaged with f3D-SIM.

To explore the role of explosive cell lysis in mediating eDNA release during the development of submerged biofilms, we examined biofilm formation by wild-type PAO1 and the endolysin mutant PAO1Δ*lys* after 8 h of culture. PAO1 produced numerous microcolony structures that stained with the eDNA stains EtHD-2 (Fig. 4b,c) or TOTO-1 (Supplementary Fig. 4a). Surface-attached round cells were also visible at this time point (Fig. 4b inset). PAO1Δ*lys* was found to be severely defective in biofilm formation and showed no microcolony structures or round cells (Fig. 4b,c). Wild-type PAO1 biofilms cultured in the presence of DNaseI, produced no microcolonies confirming a requirement for eDNA in submerged biofilm development under these assay conditions (Fig. 4b,c). The defects in microcolony development and round cell formation in PAO1Δ*lys* could be complemented with *lys* provided *in trans* (Fig. 5d; Supplementary Fig. 4b). These observations indicate that explosive cell lysis mediated via the endolysin Lys is responsible for the release of eDNA required for the formation of submerged biofilms by *P. aeruginosa*.

Interestingly, we found that the addition of exogenous *P. aeruginosa* PAO1 genomic DNA to the culture media was not able to effectively restore biofilm formation to PAO1Δ*lys* and instead significantly inhibited microcolony formation in wild-type PAO1 (Fig. 4e). These observations suggest that eDNA needs to be provided in high concentrations at the substratum to initiate biofilm formation and/or there are other components released through explosive cell lysis that are also required for the development of *P. aeruginos*a biofilms.

### Explosive cell lysis mediates MV biogenesis

Despite the importance of bacterial MVs in various processes and their ubiquitous distribution in nature, the underlying molecular mechanisms of MV biogenesis are not well understood. We noted during live-cell imaging of *P. aeruginosa* interstitial biofilms cultured in the presence of a fluorescent membrane stain, the presence of numerous highly dynamic fluorescent particles (Supplementary Movie 7). Fast three-dimensional-structured illumination super-resolution microscopy (f3D-SIM) revealed that these particles were a mixture of MVs and membrane fragments many of which were linked in chains and remained tethered to neighbouring cells (Fig. 5a; Supplementary Movie 8). We measured the sizes of 268 MVs located *in situ* in live *P. aeruginosa* biofilms that could be clearly visualized by f3D-SIM as vesicular and found these ranged in the size from 110 to 800 nm with the majority having sizes of 150–300 nm in diameter (Fig. 5b). Note, as bacterial MVs have been reported to range from 50 to 250 nm (refs 1, 2), it is likely that the membrane particles observed with f3D-SIM are MVs that are smaller than the resolution limit of this imaging technique (∼110 nm).

*P. aeruginosa* biofilms have been shown to contain MVs that interact with eDNA^3^,^22^. We used f3D-SIM to examine the localization of eDNA and MVs in live *P. aeruginosa* interstitial biofilms. This revealed that sites of eDNA release were often situated in areas that also contained abundant MVs (Fig. 5c), which suggests that the process of MV production and explosive cell lysis may be linked temporally and spatially. As we have determined that the endolysin Lys is required for eDNA release, we used f3D-SIM to determine if PAO1Δ*lys* also showed a deficiency in MV production in interstitial biofilms. We found that this strain produces very few MVs compared with wild-type PAO1 (Fig. 5d) indicating that Lys is required for both eDNA release through explosive cell lysis and MV biogenesis in interstitial biofilms.

**Figure 6:**
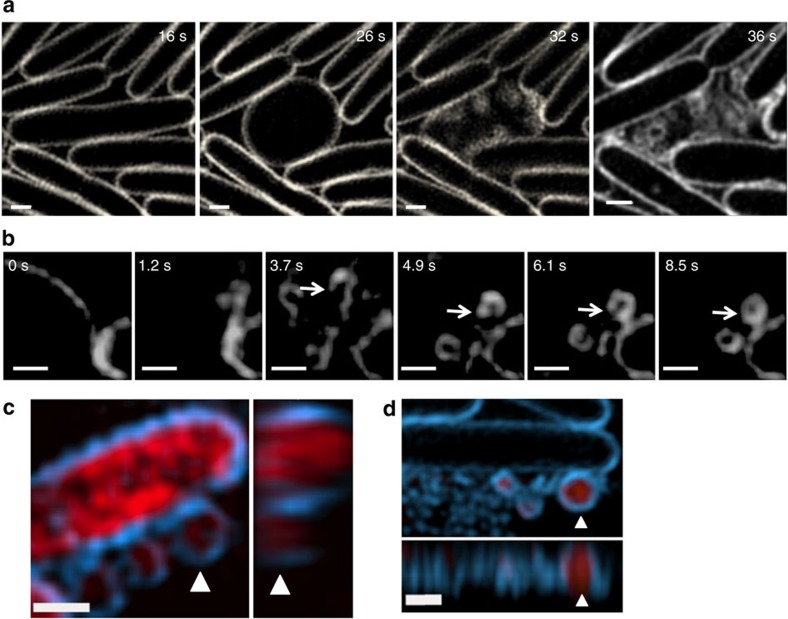
MVs are produced as a consequence of explosive cell lysis in *P. aeruginosa* biofilms. (**a**,**b**) f3D-SIM time-series of live interstitial biofilms in the presence of FM1-43FX (white). Time in seconds, top right; scale bar, 0.5 μm. (**c**) f3D-SIM of *P. aeruginosa* PAK-expressing mChFP (red) in the presence of FM1-43FX (blue). *xy* (left) and corresponding *yz* (right) views showing a large MV containing mChFP (arrow); scale bar, 0.5 μm. (**d**) f3D-SIM of live PAK interstitial biofilms in the presence of FM1-43FX (blue) and EthHD-2 (red). *xy* (upper) and corresponding *xz* (lower) views showing a large MV containing eDNA (arrow); scale bar, 0.5 μm

In Gram-negative bacteria, MVs are thought to be produced through blebbing of the outer membrane^1^. It is conceivable that the transition from rod-shaped to round cells caused by the putative peptidoglycan hydrolase activity of the Lys endolysin is concomitant with weakening of the connection between the outer membrane and the peptidoglycan of the cell wall and could therefore be associated with the release of MVs. To examine the biogenesis of MVs by *P. aeruginosa* we used f3D-SIM to follow MV production in live interstitial biofilms to ascertain if blebbing occurs during or after round cell transition. Surprisingly, f3D-SIM revealed that MVs did not appear to be formed through membrane blebbing from either rod-shaped or round cells, but instead were derived from shattered membrane fragments that were produced as a consequence of explosive cell lysis (Fig. 6a,b; Supplementary Movies 9 and 10). The rate of vesicularization of membrane fragments produced through cellular explosions was extremely rapid and often too dynamic to capture by f3D-SIM, which requires objects to be stationary within the period of each image acquisition (∼1 s). However, we were occasionally able to capture the formation of MVs that formed more slowly and found that these were formed via the curling and self-annealing of membrane fragments produced after explosive cell lysis (Fig. 6b; Supplementary Movie 10).

*P. aeruginosa* MVs have been reported to contain a variety of cellular components including DNA, peptidoglycan and proteins derived from the outer membrane, periplasm, inner membrane and cytoplasm^1^,^3^,^4^,^23^,^24^,^25^,^26^,^27^. As both cytoplasmic proteins and DNA are efficiently released into extracellular milieu at sites of explosive cell lysis (Fig. 1b,c), we hypothesize that the packaging of MV cargo could be accounted for by a mechanism in which cellular content that had been released through cell lysis is captured by membrane fragments as they self-anneal into MVs. Indeed, f3D-SIM of live *P. aeruginosa* interstitial biofilms showed that MVs in the interstitial biofilms of a *P. aeruginosa* strain that expresses cytoplasmic mCherry fluorescent protein contain this protein (Fig. 6c). Furthermore, we found that when *P. aeruginosa* interstitial biofilms were cultured in the presence of the eDNA stain EthHD-2 and visualized with f3D-SIM, MVs were observed to contain EtHD-2-stained DNA within the vesicle lumen (Fig. 6d). As EtHD-2 does not enter round cells and only interacts with eDNA after its release into the environment (Supplementary Movie 3), the presence of EthHD-2-stained eDNA within the lumen of MVs supports the idea that following the release of eDNA through explosive cell lysis, some eDNA fragments are captured during MV vesicularization.

**Figure 7:**
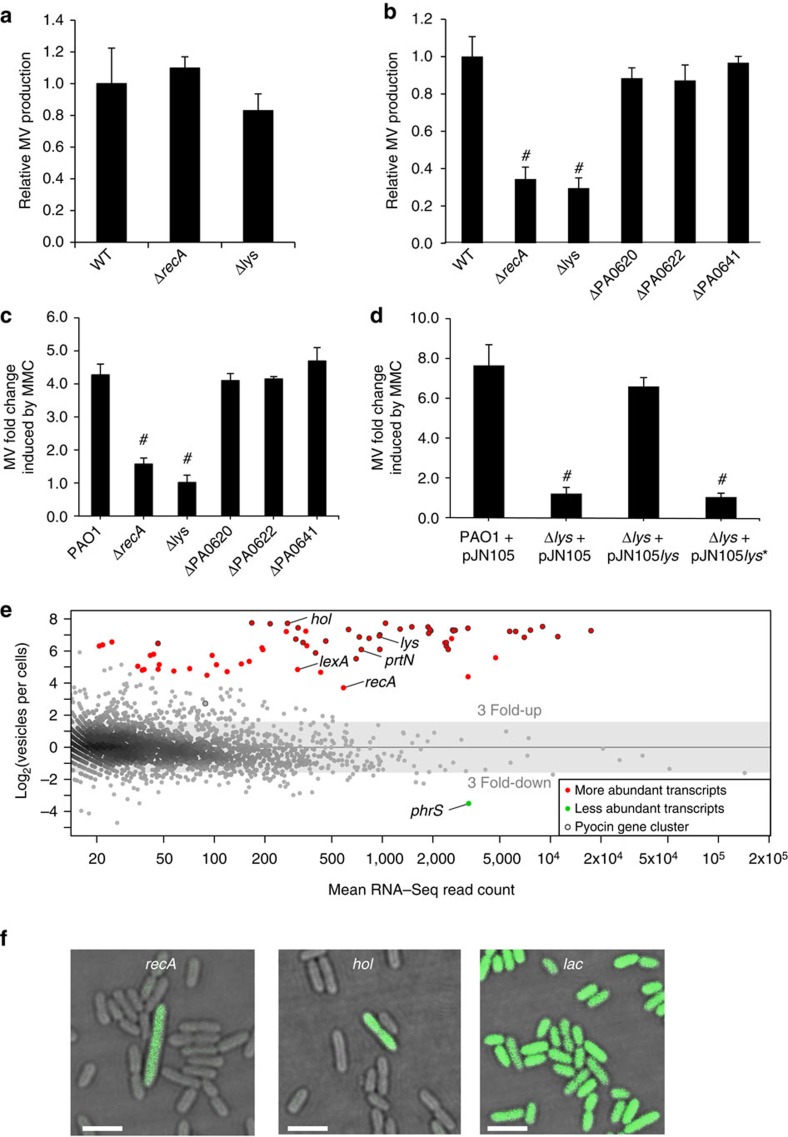
Lys is involved in stress-induced MV formation of planktonic cells. (**a**) MV production in *P. aeruginosa* PAO1 and isogenic mutants were analysed after 16 h of incubation under oxic planktonic growth conditions. *n*=3; mean±s.d. (**b**) MV production in *P. aeruginosa* PAO1 and isogenic mutants were analysed after 16 h of incubation under anoxic planktonic growth conditions. Values indicate the mean±s.d. of three replicates. *n*=3; mean±s.d. #*P*<0.001 versus wild type (WT) (Student's *t*-test). (**c**) MV production by planktonic *P. aeruginosa* PAO1 and isogenic mutants cultured in the presence of MMC (200ngmL^−1^) relative to no MMC, *n*=3; mean±s.d. #*P*<0.0005 (Student's *t*-test). (**d**) Catalytic activity of Lys is required for genotoxic stress-induced MV formation, *n*=3; mean±s.d. #*P*<0.0005 (Student's *t* test). (**e**) MA plot showing the comparison of mRNA levels associated with MVs with the transcript levels of stationary phase cells. More and less abundant transcripts in MVs are indicated by red and green dots, respectively (*P* value <0.02). Transcripts from the pyocin gene cluster (PA0610 to PA0648) are circled in black. (**f**) Promoter activities of *recA, hol* and *lacZ* (control) under non-inducing conditions were monitored by the aid of plasmids containing transcriptional fusions of the respective promoter regions to *eGFP.* Cells expressing GFP are green; scale bar, 2.5 μm.

Our observations have shown that explosive cell lysis mediated by the endolysin Lys is required for MV biogenesis in *P. aeruginosa* biofilms. We also examined the role of Lys in producing MVs in planktonic culture. Interestingly, PAO1Δ*lys* showed similar levels of MV production as the wild type under normal oxic growth in liquid medium (Fig. 7a). However, the importance of Lys in MV production was evident under conditions that stimulate MV production through induction of the SOS response such as anoxic growth or exposure to MMC^4^ (Fig. 7b,c). We found that when either *recA* or *lys* was inactivated, stress-induced MV formation was also greatly impaired whereas inactivation of pyocin structural components (pyocin tail, tail fibre or tail sheath)^4^, had no effect on MV formation (Fig. 7b,c). The defects in MMC-induced MV production by PAO1Δ*lys* could be rescued by complementation with the wild-type *lys* but not the catalytic lys* mutant allele (Fig. 7d). Promoter-reporter eGFP fusions show that *P*_*recA*_ and *P*_*hol*_ were also significantly induced by exposure to MMC (Supplementary Fig. 5).

MVs from *Escherichia coli* (*E. coli*) and *Prochlorococcus* have recently been shown to contain extracellular RNA^24^,^28^. We have found that MVs from *P. aeruginosa* also contain RNA (Fig. 7e; Supplementary Fig. 6). The presence of intact 16S and 23S rRNAs in these MVs suggests that the MV-associated RNA is not rapidly degraded (Supplementary Fig. 6). We speculated that the MV-associated mRNA may indicate the physiological state of the cells at the time the MVs were produced. A comparison of the mRNA abundances in MVs and planktonic cells obtained from oxic planktonic cultures (under non-inducing conditions) revealed that MVs are highly enriched for mRNAs that are typically expressed as part of the SOS response in *P. aeruginosa* following exposure to oxidative stress, DNA-damaging agents or antibiotics such as CPFLX^20^,^21^ (Fig. 7e; Supplementary Fig. 7e,f; Supplementary Data 1). In addition to mRNA of key regulators of the SOS response, including *lexA* and *recA*, transcripts of all genes of the R- and F-pyocin region (between PA0610 and PA0648 with the exception of PA0611) were greatly increased relative to planktonic cells. We also sequenced the DNA present in purified MVs and found that the sequences obtained covered the entire genome (Supplementary Fig. 6g), consistent with our hypothesis that eDNA fragments are captured by vesicularizing membranes.

The finding that planktonic MVs are enriched for certain mRNAs, including SOS-stress response and pyocin genes, could be explained if MVs are only produced by a small subpopulation that has induced the SOS response. In fact, heterogeneous induction of the SOS response has been reported for cultures of *E. coli*, where about 1% of the population was reported to be induced^29^. Promoter–reporter eGFP fusions of *P*_*hol*_ or *P*_*recA*_ and genomic transcriptional fusions revealed that under standard (non-inducing) planktonic growth conditions only a small fraction of the cells (<1%) showed strong fluorescence, while a fusion of the constitutive *lac* promoter to *eGFP* was expressed in the large majority of cells (Fig. 7f; Supplementary Fig. 7). Hence, even under optimal planktonic growth conditions a small proportion of the cells stochastically induce the SOS response and pyocin expression.

**Figure 8:**
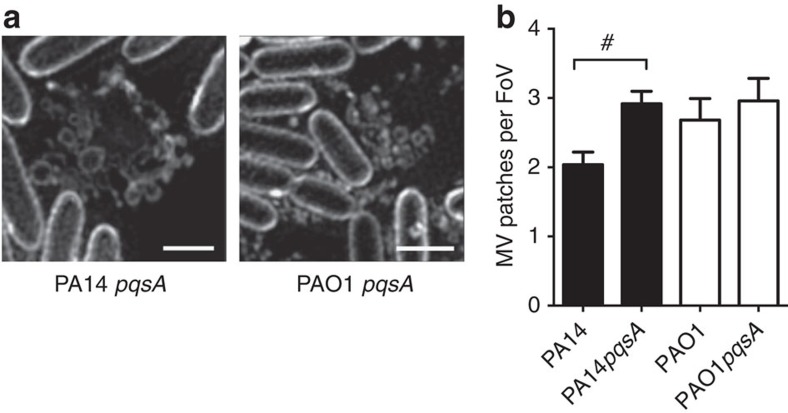
PQS is not required for MV production in interstitial biofilms. (**a**) f3D-SIM of PA14*pqsA* and PAO1*pqs*A biofilms cultured in the presence of FM1-43FX (white) showing MV patches; scale bar, 1 μm. (**b**) Quantification of MVs in random fields of view (40 μm × 40 μm) of PA14 (*n*=54), PA14*pqsA* (*n*=60), PAO1 (*n*=22) and PAO1*pqsA* (*n*=24); #*P*<0.0001, unpaired *t*-test with Welch's correction.

We have found that explosive cell lysis is associated with the production of MVs in interstitial biofilms and planktonic cultures of *P. aeruginosa*. The *Pseudomonas* Quinolone Signal (PQS) has been reported to be crucial for MV production in *P. aeruginosa*^30^; whereas a number of other studies have found that PQS is not required for MV production in planktonic cultures under stressed or unstressed conditions^31^,^32^,^33^. Therefore, to further explore the contribution of PQS to MV biogenesis we analysed MV production in interstitial biofilms of *pqsA* mutants which do not produce PQS^34^. These assays showed that the *pqsA* mutants of *P. aeruginosa* strains PAO1 and PA14 were not defective in the production of MVs in interstitial biofilms relative to their isogenic parent strains (Fig. 8). In fact PA14*pqsA* produced significantly more MVs than its isogenic parent in this assay. Furthermore, our RNA-seq analyses of MVs obtained from unstressed planktonic cultures revealed that the small RNA PA3305.1 (PhrS), which has been shown to stimulate *Pseudomonas* quinolone signal (PQS) production^35^, is less abundant in planktonic MVs than planktonic cells. This is consistent with PQS not having a critical role in the production of MVs with RNA cargo which have presumably been derived through explosive cell lysis. These observations indicate that explosive cell lysis-mediated MV production in biofilms and planktonic cultures is independent of PQS.

## Discussion

In this study, we have shown that explosive cell lysis accounts for the efficient liberation of a variety of cellular components including cytosolic proteins, eDNA and MVs that may serve as public goods in *P. aeruginosa* biofilms. We have determined that explosive cell lysis in *P. aeruginosa* is due to an endolysin-encoding gene (*lys*) which is located on the genome within a cryptic prophage gene cluster that encodes the R- and F-type pyocins. Expression of genes in the R- and F-pyocin gene cluster, including *lys*, is known to be upregulated by exposure to exogenous stresses through the RecA-dependent SOS response^12^,^17^,^18^,^19^,^20^,^21^ and we found that explosive cell lysis and *lys* expression is induced by exposure to exogenous stresses and that this is regulated through RecA. However, it is yet to be determined if explosive cell lysis under non-stress conditions is a programmed cell death pathway induced by fratricide or altruistic suicide^36^. We found that *P. aeruginosa* Δ*recA* strains were significantly abrogated in explosive cell lysis events even in the absence of exogenous stress, which suggests that there may be endogenous cues that induce RecA-mediated *lys* expression. Endogenous stress that activates RecA is known to occur in *P. aeruginosa* biofilms^37^. Furthermore, the RecA-mediated SOS stress response can lead to a block in cell division in some bacteria^38^ and we observed that many of the cells that go on to become round cells appeared to be blocked in cell division. Therefore it is possible that endogenous stress may act as a trigger to stimulate Lys-mediated explosive cell lysis. Alternatively, explosive cell lysis in *P. aeruginosa* may be a consequence of stochastic expression of the pyocin gene locus (including *lys*) to enable release of pyocins from the cell. Interestingly, we found that the pyocin structural genes are not required for explosive cell lysis to occur. Therefore, it is possible that *lys* may also be expressed independently of the structural genes of the R- and F-pyocin locus and that explosive cell lysis can occur independently of pyocin release. Whatever the mechanism by which explosive cell lysis is triggered, it is clear from our observations that this process liberates public goods including eDNA, cytosolic content and MVs that can be exploited by other cells. Indeed, we found that populations of *lys* mutants that are unable to undergo explosive cell lysis are severely abrogated in biofilm formation.

Also encoded in the R- and F-pyocin gene cluster is the putative holin, Hol (PA0614)^17^, overexpression of which has been shown to cause increased lysis of *P. aeruginosa*^39^. While we have not examined the contribution of Hol in explosive cell lysis in this study, we expect that Hol facilitates translocation of Lys across the inner membrane to degrade the cell wall peptidoglycan leading to explosive cell lysis. Indeed, here we showed that *in trans* expression of both *hol* and *lys* were required to elicit cell lysis in *E. coli* (Supplementary Fig. 2) indicating that Hol facilitates translocation of Lys across the *E. coli* inner membrane. However, as other putative *P. aerugino*sa holins CidA^40^ and AplB^41^ have previously been reported to be associated with lysis of *P. aeruginosa* cells, it is possible that these may also contribute to translocation of Lys across the inner membrane.

The R- and F-pyocin gene cluster is part of the *P. aeruginosa* accessory genome as it is not present in all strains^42^. However, we found that *lys* is highly conserved in all complete genomes of *P. aeruginosa* strains available in the public genome databases (EMBL/GenBank/DDBJ), with 88–100% nucleotide identity over the full length of the 630 bp gene. It is likely that other prophages also encode the endolytic activity required for explosive cell lysis. Furthermore, genes with high similarity to *lys* were also found in the genomes of other *Pseudomonas* species and many other bacterial genera indicating that this might be a conserved phenomenon in bacteria.

Using live-cell super-resolution microscopy, we have determined that explosive cell lysis is a mechanism for bacterial MV biogenesis and MV cargo packaging. We found that the vesicularization of shattered membrane fragments that are produced by exploding bacteria are likely to capture cellular components released into the extracellular milieu as they self-anneal into MVs. To our knowledge, this is the first direct observation in live bacterial cells of either MV biogenesis or the process by which cellular content is liberated into the extracellular matrix of bacterial biofilms. Although MV biogenesis via explosive cell lysis is unlikely to control the cargo of MVs precisely, this mechanism provides a convincing explanation as to why cytoplasmic material such as DNA, RNA and cytoplasmic proteins are present in MVs. Interestingly, we found that *lys* mutants were significantly reduced in MV production in biofilms and in stressed planktonic cultures indicating that most MVs are produced through explosive cell lysis events under these conditions. In contrast, however, we found that *lys* mutants were not defective in MV production in non-stressed planktonic cultures, which suggests that other mechanisms such as outer membrane blebbing may account for the majority of MV biogenesis under these conditions. However, as our RNA-Seq data was obtained from MVs obtained from non-stressed planktonic cultures and showed upregulation of SOS response genes including *recA* and *lys*, it is likely that explosive cell lysis also accounts for the production of some MVs that contain cytoplasmic content (including RNA) in non-stressed planktonic cultures. Indeed, during our submerged biofilm formation assays, we observed explosive cell lysis events in the planktonic phase.

Taken together, our data demonstrate a novel role for bacteriophage-associated endolysins in MV biogenesis. These results not only imply that the ability to produce MVs can be conferred via bacteriophages as a consequence of the bacteriophage lytic cycle but also may explain why MVs can harbour complete viral genomes^43^ and DNA associated with MVs isolated from open ocean samples are strongly enriched for viral sequences^28^,^44^.

As many species of bacteria and archaea produce MVs, moonlighting proteins and/or a biofilm matrix comprised of eDNA, lipids and cytoplasmic proteins^1^,^6^,^8^,^45^, and prophage and prophage-like elements are a common feature of bacterial genomes^46^, phage-mediated explosive cell lysis may be a ubiquitous mechanism for the production of MVs and release of cytosolic public goods in bacterial biofilms. At low levels, phage-mediated explosive cell lysis is likely to be beneficial to bacterial communities through the provision of a mechanism for the efficient release of cell-derived public goods. Indeed, prophage and prophage-like elements have been associated with increased fitness of bacterial populations^12^ and the endolysin genes of cryptic prophages appear to experience purifying evolution, which suggests that they are under positive selection pressure^47^.

## Methods

### Bacterial strains and plasmids

*P. aeruginosa* and *E. coli* strains used in this study, as well as plasmids, are listed in Supplementary Table 1. The primers used in this study are listed in Supplementary Table 2. Gene deletion mutants and genomic *eGFP* transcriptional fusions were carried out using pG19II (ref. 48) for homologous recombination as previously described^49^. The pG19II-derived plasmids were transferred into *P. aeruginosa* strains through conjugation with *E. coli* S17-1. The mutants were analysed by PCR. PA0629 fused with six histidine residues was cloned into the expression vector pJN105 by using cPA0629H_F/cPA0629H_R primer pairs to amplify this region of the PAO1 chromosome. PA0614 was cloned in to the expression vector pET21b by using PA0614b_F/PA0614b_R primer pairs to amplify the PAO1 chromosome. PA0629* was constructed by overlap extension PCR by inserting a point mutation that replaced the catalytic glutamic acid (E51) with valine. For the first round PCR, the PAO1 chromosome was amplified with PA0629AA_F/PA0629_E51V_R or PA0629_E51V_F/cPA0629H_R primer sets. The PCR products were mixed for overlap extension PCR^50^. Finally, the full-length PA0629* was amplified with cPA0629H_F/cPA0629H_R and cloned into pJN105. Point mutation was confirmed by sequencing (Hokkaido System Science, Japan). The catalytic site was predicted by the Phyre server^51^. The *recA* promoter region was introduced into pMEXGFP, by amplifying this region of the PAO1 genome with pRecA_F/pRecA_R primer pairs as previously described^32^. The *lac* promoter was amplified from pUC19 with pLac_F/pLac_R primers and inserted into pMEXGFP, to construct the constitutive eGFP expression vector, pMLAC-G. The plasmid pUCP*mChFP* was constructed by PCR amplifying the *mChFP* gene from the template pmCherry-C2 (CLONTECH Laboratories, Inc., Palo Alto, CA, USA) using the primer pair mCherry_F/mCherry_R and cloning the PCR fragment into the *Sph*I and *Hin*dIII sites of pUCPKS.

### Growth conditions

*P. aeruginosa* and *E. coli* were cultured in either cation-adjusted Mueller Hinton broth (CAMHB) or Luria-Bertani (LB) broth for *P. aeruginosa* or LB broth for *E. coli* or on LB agar (1.5%) and incubated at 37 °C. Antibiotic concentrations used for selection of *E. coli* were 100 μg ml^−1^ ampicillin, 10 μg ml^−1^ gentamicin and 10 μg ml^−1^ tetracycline and for *P. aeruginosa* were 250 μg ml^−1^ carbenicillin and 100 μg ml^−1^ gentamicin. Fluorescent stains used in this study (obtained from Life Technologies) were the eDNA stain TOTO-1 iodide (1 μM), the lipophilic membrane stain FM1-43FX (5 μg ml^−1^), and the eDNA and dead cell stain ethidium homodimer-2 (EthHD-2; 1 μΜ). DNaseI (D5025, Sigma) was used at 100 Kunitz units per ml. For MV assays, *P. aeruginosa* was grown aerobically at a starting optical density of 0.01 at 600 nm (OD_600_), unless otherwise specified. For *P. aeruginosa* anoxic cultures, LB medium was supplemented with 100 mM KNO_3_, in butyl-rubber sealed Hungate tubes and the head space was replaced with argon by flushing gas through a needle^49^. No growth was observed under the anoxic condition, when KNO_3_ was not added in the medium or when a nitrate reductase mutant was inoculated, confirming that the growth was dependent on denitrification. MMC and CPFLX were used to induce MV production at concentrations indicated in the figure legends. **L**-Arabinose was added at concentrations indicated in the figure legends to induce gene expression under the control of *araBAD* promoter on pJN105.

### Biofilm assays

Interstitial biofilm assays were performed as described previously^7^. Briefly, microscope slides were coated in nutrient media solidified with gellan gum (TMGG; 0.4 × LB, 0.1% MgSO_4_·7H_2_O, 0.8% GelGro gellan gum (MP Biomedicals, Santa Ana, CA, USA)) and where indicated fluorescent stains were added to the molten media immediately prior to pouring. Once set, the TMGG slab was inoculated with a small amount of overnight plate culture, a coverslip applied and incubated at 37 °C for 4–6 h prior to microscopic imaging. Filter disc diffusion assays were performed as described previously^52^ with filter discs saturated in CPFLX (100 μg ml^−1^), MMC (500 μg ml^−1^) or sterile water. Briefly, 75 μl of the test solution was applied to a filter disc (Whatman 6 mm, GE Healthcare). Each disc was dried for 2 h and then applied to a TMGG-coated microscope slide and a gradient allowed to establish for 1 h and the disc removed. The TMGG was inoculated with the strain of interest 5 mm from the disc, a coverslip applied and incubated at 37 °C for 4 h.

To assay the formation of submerged biofilms, overnight cultures were washed three times in fresh CAMHB, diluted to an equivalent of 1/100 in CAMHB and cultured for 2 h at 37 °C with shaking (250 r.p.m.). The cultures were then transferred to an eight-well IBIDI-treat chamber slide (IBIDI GmbH, Germany) and incubated statically at 37 °C. To examine eDNA release during the initial stages of biofilm formation, the biofilm culture media included the eDNA stain TOTO-1 and time-lapse imaging (DV Elite; × 100 objective) commenced after 1 h static culture. To visualize biofilm formation after 8 h static culture, wells were washed twice with fresh media. CAMHB containing eDNA stain was added to the wells and biofilms and cells at the substratum imaged with phase contrast and wide-field fluorescence microscopy (Olympus IX71, × 100 objective). To assess influence of exogenous eDNA on formation of submerged biofilms, *P. aeruginosa* PAO1 chromosomal DNA was purified (MasterPure DNA purification kit, Epicentre) and added to CAMHB at 1 μg ml^−1^.

### MV isolation and quantification

Cell culture was centrifuged for 10 min at 15,000*g*, 4 °C, and the supernatant filtered through a 0.4 μm pore size polyvinylidene difluoride filter (Merck Millipore, Germany). The supernatant was ultracentrifuged for 1 h at 150,000*g*, 4 °C, and the pellet resuspended in double distilled water for MV quantification and in Optiprep (AXIS-SHIELD, Scotland) for further purification^32^. For further MV purification, the MV containing pellet was suspended in 45% iodixanol (Optiprep) and purified by density gradient ultracentrifugation as previously described^4^. Briefly, the MV suspension in 45% iodixanol was layered with 40, 35, 30, 25 and 20% iodixanol. Iodixanol was prepared in 10 mM HEPES/0.85% NaCl. Gradients were ultracentrifuged at 100,000*g* for 3 h and fractions were removed from the top. MVs were quantified by staining with FM4-64 fluorescent dye (Life Technologies, USA)^4^. FM4-64 shows low fluorescence in water but fluoresces intensely on binding to the membrane. Fluorescence (558 nm excitation/734 nm emission) was quantified using Varioscan flash fluorometer (Thermo Scientific, USA)^32^.

### Illumina sequencing of RNA and DNA extracted from MVs

Total RNA from stationary phase PAO1 cells and MVs grown aerobically in LB medium was isolated using a modified hot acid phenol protocol^53^. MVs were separated from the cells as described above. Genomic DNA was removed by DNaseI (Promega, USA) treatment and the resulting RNA quality was examined for DNA contamination by PCR (40 cycles). After checking the quality of the RNA using RNA Nano Chips (Agilent 2100 Bioanalyzer), 10 μg from each total RNA sample was used for first and second strand complementary DNA synthesis according to the TruSeq RNA preparation guide (Illumina, USA). DNA was extracted from purified MVs with ISOPLANT II (Nippongene, Japan), according to the manufacturer's instructions. Double-strand cDNA and fragmented MV DNA libraries were prepared for sequencing according to the manufacturer's instructions (TruSeq DNA preparation guide, Illumina). Single-end 100 nucleotide sequence reads were obtained using the Illumina HiSeq2000 sequencer and processed with Casava version 1.8. Sequencing reads were mapped to the PAO1 genome (RefSeq NC_002516.2) using CLC Genomics Workbench v4.9 (CLCbio, Denmark) allowing up to two mismatches per read. We only considered genes whose expression level in one of the samples (whole cells, MVs) was above 0.2 RPKM (reads per kilobase per million mapped reads) and explicitly excluded rRNA genes (3498 of 5682 genes, that is, 62%, satisfied these criteria). To detect differentially abundant transcripts, RNA-Seq count data (unambiguously mapped reads) were subsequently analysed for differential abundance in MVs against the whole cell transcripts employing the R package DESeq (version 1.6.1)^54^. Transcripts with resulting *P* values of <0.02 were considered as differentially abundant. Fold changes in the transcript abundance (log-scale) were visualized with respect to mean signal intensity (RNA-seq read count) using R as described^55^. DNA abundance levels (log(RPKM)) were visualized with respect to genomic location using DNAplotter^56^.

### Verification of RNA-Seq data by qPCR

The expression of PAO1 genes PA0610 (*prtN*), PA0617, PA0629, PA0985 (*pyoS5*), PA1150 (*pys2*), PA3007 (*lexA*) and PA0576 (*rpoD*) was analysed by quantitative PCR with reverse transcription (qRT–PCR) using Brilliant III Ultra-Fast SYBR Green QPCR Master Mix (Agilent, Switzerland) and an Mx3000P instrument (Agilent). cDNA was prepared from biological replicates as previously described^57^. Briefly, total RNA was isolated by hot-phenol extraction^53^, genomic DNA removed by DNaseI (Promega, USA). RNA was further purified using the RNeasy Kit (Qiagen) and cDNA synthesized using M-MLV Reverse Transcriptase, RNase H Minus (Promega). Each PCR reaction was run in triplicate containing three dilutions of cDNA (15, 7.5 and 3.75 ng), 12.5 μl of 2 × Brilliant III Ultra-Fast SYBR Green QPCR Master Mix and 0.7 μM of individual primers in a total volume of 25 μl. Fold changes in expression were calculated using the ΔΔ CT method^58^. The primary σ factor gene *rpoD* was used as a reference for normalization. The primers used are listed in Supplementary Table 2.

### Promoter activity assay

The promoter assay was performed with the *eGFP* reporter plasmid pMEXGFP as previously described with the following modifications^32^. eGFP expression was measured with a fluorometer (Varioscan flash, Thermo Scientific) with emission at 488 nm and excitation at 509 nm, and was normalized to cell growth (OD_600_).

### Western blotting

Cells were washed once in 50 mM Tris-HCl buffer (pH 8.0) and resuspended in the same buffer. The resuspended cells were sonicated, mixed with equal amount of SDS–polyacrylamide gel electrophoresis (SDS–PAGE) sample buffer and incubated at 95 °C for 5 min. An amount of 2.5 μg protein of each sample was loaded to 12% SDS–PAGE gels and transferred on a polyvinylidene difluoride membrane by electroblotting. The membrane was blocked with 2.5% (w/v) skim milk in TBS-T buffer (Tris-bufferd saline (pH 7.5) containing 0.05% Tween 20) for 1 h at room temperature. The membrane was washed twice with TBS-T buffer and incubation for 1 h with anti-His tag antibody (MBL, Japan) diluted 1:1,000 in Can Get Signal solution I (TOYOBO, Japan). After washing the membrane twice in TBS-T buffer, the membrane was incubated for 20 min with a secondary anti-mouse antibody conjugated with horseradish peroxidase (GE Healthcare, USA) at a dilution of 1: 5,000 in Can Get Signal solution II (TOYOBO, Japan). The membrane was washed twice in TBS-T buffer and exposed to the substrates in ImmunoSTARLD (Wako, Japan). Bands were visualized with a C-DiGit Blot Scanner (LI-COR, USA).

### Microscopy

Phase contrast and wide-field fluorescence microscopy was performed using an Olympus IX71 inverted research microscope with a × 100 1.4 numerical aperture UPlanFLN objective, FViewII monochromatic camera and AnalySIS Research acquisition software (Olympus Australia, Notting Hill, VIC, Australia) fitted with an environmental chamber (Solent Scientific, Segensworth, UK); a Nikon Ti inverted research microscope with a × 100 1.45 numerical aperture PlanApo objective, NIS Elements acquisition software (Nikon Instruments, Tokyo, Japan), solid state illumination (Lumencor, Beaverton, OR, USA), Cascade 1Kx1K EMCCD camera (Photometrics) and fitted with an environmental chamber (ClearState Solutions, Mt Waverley, VIC, Australia); or a DeltaVision Elite inverted research microscope with a × 100 1.4 numerical aperture UPlanFLN objective, InsightSSI illumination, SoftWorX acquisition software, fitted with a WeatherStation environmental chamber (Applied Precision, GE Healthcare, Issaquah, WA, USA) and a scientific CMOS 15-bit camera (pco.edge, PCO AG, Kelheim, Germany).

f3D-SIM was performed with a V3 DeltaVision OMX 3D-SIM system fitted with a Blaze module (Applied Precision, GE Healthcare, Issaquah, USA). Solid-state lasers provided wide-field illumination and images were captured using a × 60 1.4 numerical aperture UPlanSApo objective (Olympus, Toyko Japan), standard filter sets and a scientific CMOS 512 × 512 pixel 15-bit camera (pco.edge, PCO AG, Kelheim, Germany). Interference patterns were generated by interfering light beams^59^ and samples were sectioned using a 125-nm Z-step size. Raw 3-phase images were then reconstructed to extract finer detail using the Gustafsson algorithms^60^,^61^. Wide-field imaging was also performed using conventional mode on the DeltaVision OMX system and images were deconvolved using SoftWorX software (Applied Precision, GE Healthcare).

DIC and fluorescence microscopy was performed on an LSM710 (Carl Zeiss, Germany) mounted with a charged-couple device (CCD) camera and the Axiovision system used for operation. Confocal laser scanning microscopy was performed with a DM5500Q microscope (Leica, Germany) with Leica Application suite. Purified MVs were treated with 3 U 5 μl^−1^ of RNase A (Sigma-Aldrich, Switzerland) and stained with SYTO RNASelect green fluorescent cell stain (Life Technologies), according to the manufacturer's instructions. MVs were observed on a 1% agarose pad supplemented with 0.5 M sucrose. eGFP-expressing cells were observed on a 1% agarose pad if not specified in the figure legend.

For transmission electron microscopy, purified MVs were stained with uranyl acetate as previously explained^4^ and inspected by a JOEL JEM 2000EX transmission electron microscope by an outside facility (Hanaichi Ultrastructure Research Institute, Japan).

### Image analysis

Wide-field, deconvolved or f3D-SIM images were rendered and presented using IMARIS software (v7.7 or above, Bitplane Scientific) or FIJI^62^. Final images were prepared in Photoshop (Adobe Systems, San Jose, CA, USA). Movies were prepared using FIJI^62^. Linear adjustments to signal contrast and brightness were made in the images presented but no gamma settings were changed.

For quantitative analysis of eDNA release sites in interstitial biofilms, series of overlapping images spanning the outermost leading edge through to the main colony were obtained (Nikon Ti; × 100 objective) and stitched using the NIS Elements acquisition software (Nikon Instruments). The numbers of eDNA release sites across the monolayer region of each interstitial biofilm were quantified manually and the area of the biofilm comprised of cells identified by auto-thresholding using FIJI^62^.

To analyse the frequency of microcolonies in submerged biofilms, random images of the substrate surface were obtained (Olympus IX71; × 40 objective) and ‘Particles' (microcolonies) >100 μm^2^ identified by auto-thresholding using FIJI^62^.

Quantitative assessment of cell morphotypes in interstitial biofilms was performed using an in-house program, BacFormatics v0.7 (source code available at https://github.com/ithreeMIF/BacFormatics), that we have developed in MATLAB (The MathWorks Inc., Natick, MA, USA). BacFormatics is based on the open-source TACTICS Toolbox^63^,^64^. The following image analysis pipeline in BacFormatics was utilized to analyse 16-bit phase-contrast images. Pixel intensities of each image were inverted (*I*=65,535−*I*). To segment individual bacteria we integrated an edge-detection algorithm and enhanced the background between the cells. This includes morphological operations to connect and close the spaces between the cells, which has a net-like closed structure and is useful to split touching cells. To detect cell perimeters we used Laplacian of Gaussian (LoG) edge-detection as described previously^65^. The LoG edge-detection provides closed contour curves, which are filled and represent segmented bacteria. To accurately segment the net, we applied multiple structuring element centre surround top-hat transformation^66^. Briefly, multiple structuring elements were applied to enhance linear regions at different directions, followed by intensity thresholding to reconstruct the image. A morphological close operation was applied to each image to close spaces in the morphology of the cells (to smooth the edges)^67^. This step was followed by a background subtraction, which was applied by removing the mean intensity of the background from each pixel in the image. Cells were segmented by intensity threshold, where the cutoff level was manually adjusted or automatically chosen by Otsu's method^68^. Following the segmentation step, small segments (less than 80 pixels) and large segments (more than 3,000 pixels) were removed. Intensity pixels with zero values within the cells (holes) were converted to values of one. To detect cell clusters, the curvature and junctions of the cells were split using an algorithm written by He and Yung^69^, then the watershed algorithm was applied to separate segments that were classified as touching objects. To split two overlapping cells we trained a data set with 100 touching cells and utilized the MATLAB function classify from the Statistics Toolbox. The classifier is based on morphology parameters and ratio between concave borders at the intersection of two touching cells as shown previously^70^. Categorization of cells as round or rod morphotypes was based on circularity criteria calculated by the standard formula:


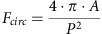


where *A* is the area and *P* is the cell perimeter. Cells with a circularity factor >0.8 were labelled as a round morphotype and <0.8 as a rod morphotype. Manual inspection was applied using a dedicated user interface that colour-labelled round cells. BacFormatics requires MATLAB R2012b version 8.0 (The MathWorks, Inc.) or later versions with the presence of the Statistics Toolbox and MATLAB Image Processing Toolbox (IPT). The BacFormatics analysis in this paper was performed on a Dell Latitude E5540 with 16 GB RAM and Intel(R) Core(TM) i7-4600U CPU 2.10GHz.

## Additional information

**Accession codes:** The RNA-Seq and DNA-Seq raw data have been deposited in the GEO database with accession code GSE63388.

**How to cite this article:** Turnbull, L. *et al*. Explosive cell lysis as a mechanism for the biogenesis of bacterial membrane vesicles and biofilms. *Nat. Commun.* 7:11220 doi: 10.1038/ncomms11220 (2016).

## Supplementary Material

Supplementary Information Supplementary Figures 1-7, Supplementary Tables 1-2 and Supplementary References

Supplementary Data 1 List of differentially abundant RNAs in MVs against planktonic cells.

Supplementary Movie 1 Explosive cell lysis releases eDNA. A *P*. aeruginosa PAK interstitial biofilm cultured on media containing the cell impermeant DNA stain TOTO-1 (green) was imaged using phase contrast and widefield fluorescence microscopy (Nikon Ti). An individual rod cell rapidly transitions to a round-shaped cell and subsequently explodes releasing eDNA. Time is shown in minutes and seconds.

Supplementary Movie 2 Long-lasting round cells are malleable. A *P*. aeruginosa PAK interstitial biofilm cultured on media containing the membrane stain FM1-43FX was imaged using widefield fluorescence microscopy (DeltaVision OMX conventional mode). An individual round cell (upper right) is shown over a period of approximately six minutes as it as it is pushed by surrounding cells and takes on a variety of shapes. When the neighboring cells move away it returns to a default spherical shape. Time is shown in minutes and seconds. A slight jump in frames is seen at 3:44 where the capture was paused for re-focusing.

Supplementary Movie 3 Round cells are viable until explosive cell lysis. A *P*. aeruginosa PAK interstitial biofilm cultured on media containing the cell impermeant DNA stain EtHD-2 was imaged using phase contrast (left panel) and fluorescence (right panel) microscopy (Nikon Ti). All cells, including two round cells, exclude EtHD-2 indicating they have intact membranes. One round cell explodes and eDNA (red) is then observed. The remaining round cell continues to exclude the EtHD-2. Time is shown in minutes and seconds.

Supplementary Movie 4 Explosive cell lysis occurs in the absence of fluorescent stains and fluorescence microscopy. A *P*. aeruginosa PAK interstitial biofilm was imaged using phase contrast microscopy (Olympus IX71) in the absence of input fluorescence excitation energy, antibiotics or fluorescent stains in the culture media. The 2 time-series show single rod cells rapidly transitioning to the round cell morphotype and then exploding. Time is shown in minutes and seconds.

Supplementary Movie 5 Exploding bacteria express pyocin genes. An interstitial biofilm of *P*. aeruginosa PAO1 containing the P_hol_-eGFP promoter reporter pM0614-G was imaged using phase contrast (left panel) and fluorescence (right panel) microscopy (Nikon Ti). The cell that rounds up and explodes expresses GFP.

Supplementary Movie 6 Explosive cell lysis occurs during early stages of submerged biofilm development. Time series of the initial stages of *P*. aeruginosa PAO1 biofilm development 1 h after inoculation showing attachment of a rod cell, its transition to round cell morphotype and subsequent explosion releasing eDNA (TOTO-1, green). Time in min (top right), scale bar 5 μm.

Supplementary Movie 7 Dynamic lipid particles in interstitial biofilms. An interstitial biofilm of *P*. aeruginosa PAK cultured on media containing the lipid stain FM1-43FX was imaged using widefield fluorescence microscopy (DeltaVision OMX conventional mode). Dynamic lipid particles can be observed bouncing amongst the migrating cells of the biofilm. Time is shown in minutes and seconds.

Supplementary Movie 8 MVs in *P*. aeruginosa interstitial biofilms. A *P*. aeruginosa PAK biofilm cultured on media containing the lipid stain FM1-43FX was imaged using f3D-SIM (DeltaVision OMX Blaze). MVs of varying diameters can be seen surrounding the cells of the biofilm and are very mobile. Chains of MVs can be seen stretching between cells. MVs are also observed squashed between closely abutted cells (see Fig. 2). Time is shown in minutes and seconds.

Supplementary Movie 9 Explosive cell lysis produces MVs. A *P*. aeruginosa PAK biofilm cultured on media containing the lipid stain FM1-43FX was imaged using f3D-SIM (DeltaVision OMX Blaze). A single rod cell transitions to a round-shaped cell and then subsequently explodes. MVs are observed immediately following the explosion and are not observed to bleb from the cell prior to explosion (see Fig.4a). Time is shown in bottom right corner in seconds and milliseconds.

Supplementary Movie 10 MVs are formed through curling of membrane fragments produced by explosive cell lysis. A *P*. aeruginosa PAK biofilm cultured on media containing the lipid stain FM1-43FX was imaged using f3D-SIM (DeltaVision OMX Blaze). A single rod cell transitions to a round-shaped cell and explodes (left panel). A magnified view of the boxed area (right panel) shows the release of shattered membrane fragments which curl and vesicularise into MVs. These newly formed MVs then come to rest on the surface of a neighboring cell. Time is shown in bottom right corner in seconds and milliseconds.
