# Supplementary material for: Explosive cell lysis as a mechanism for the biogenesis of bacterial membrane vesicles and biofilms
Source: Nat Commun. 2016 Apr 14;7:11220. doi: 10.1038/ncomms11220 (PMC4834629; doi:10.1038/ncomms11220)
Supplement: Supplementary Information — Supplementary Figures 1-7, Supplementary Tables 1-2 and Supplementary References [file ncomms11220-s1.pdf]

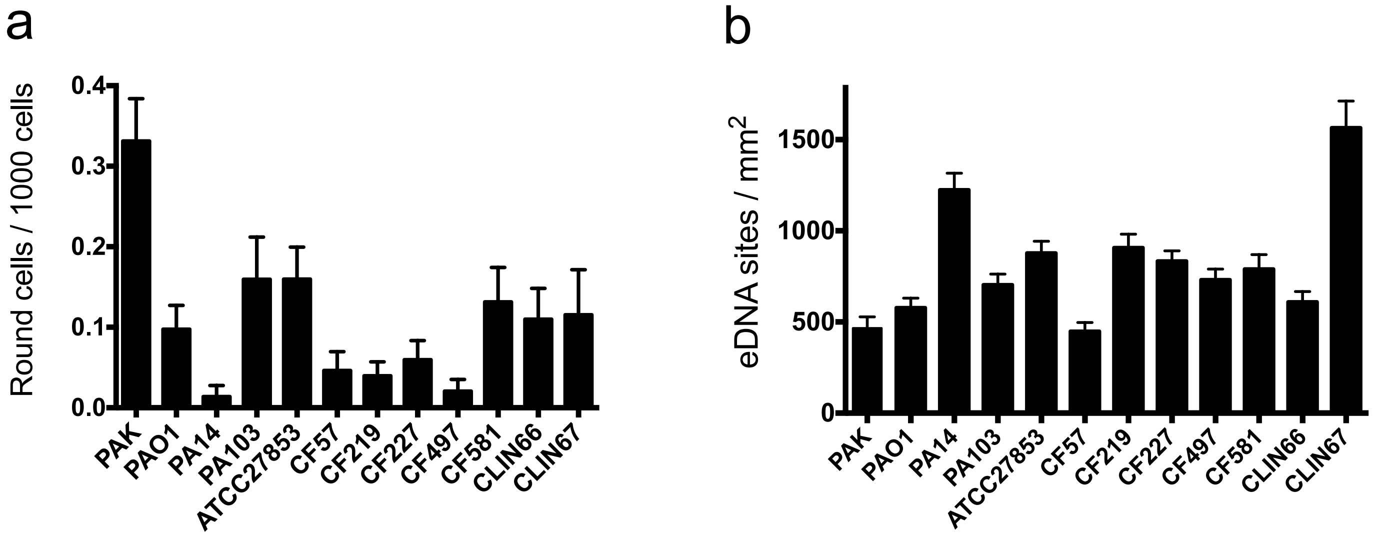

Supplementary Figure 1. Explosive cell lysis is a conserved phenotype in *P. aeruginosa* (a) Proportions of cells with round cell morphotypes in interstitial biofilm monolayers of laboratory and clinical *P. aeruginosa* strains. Computer vision was used to identify cells in 60 random images of each strain of *P. aeruginosa* (except PAK, 111 images) and characterize these as having either rod or round morphotypes. The total number of cells detected for each strain was; PAK 161628, PAO1 114559, PA14 118997, PA103 89323, ATCC27853 94866, CF57 122407, CF219 116836, CF227 98526, CF497 97103, CF581 105114, CLIN66 91460, CLIN67 57126. (b) eDNA sites in interstitial biofilms of *P. aeruginosa* strains.

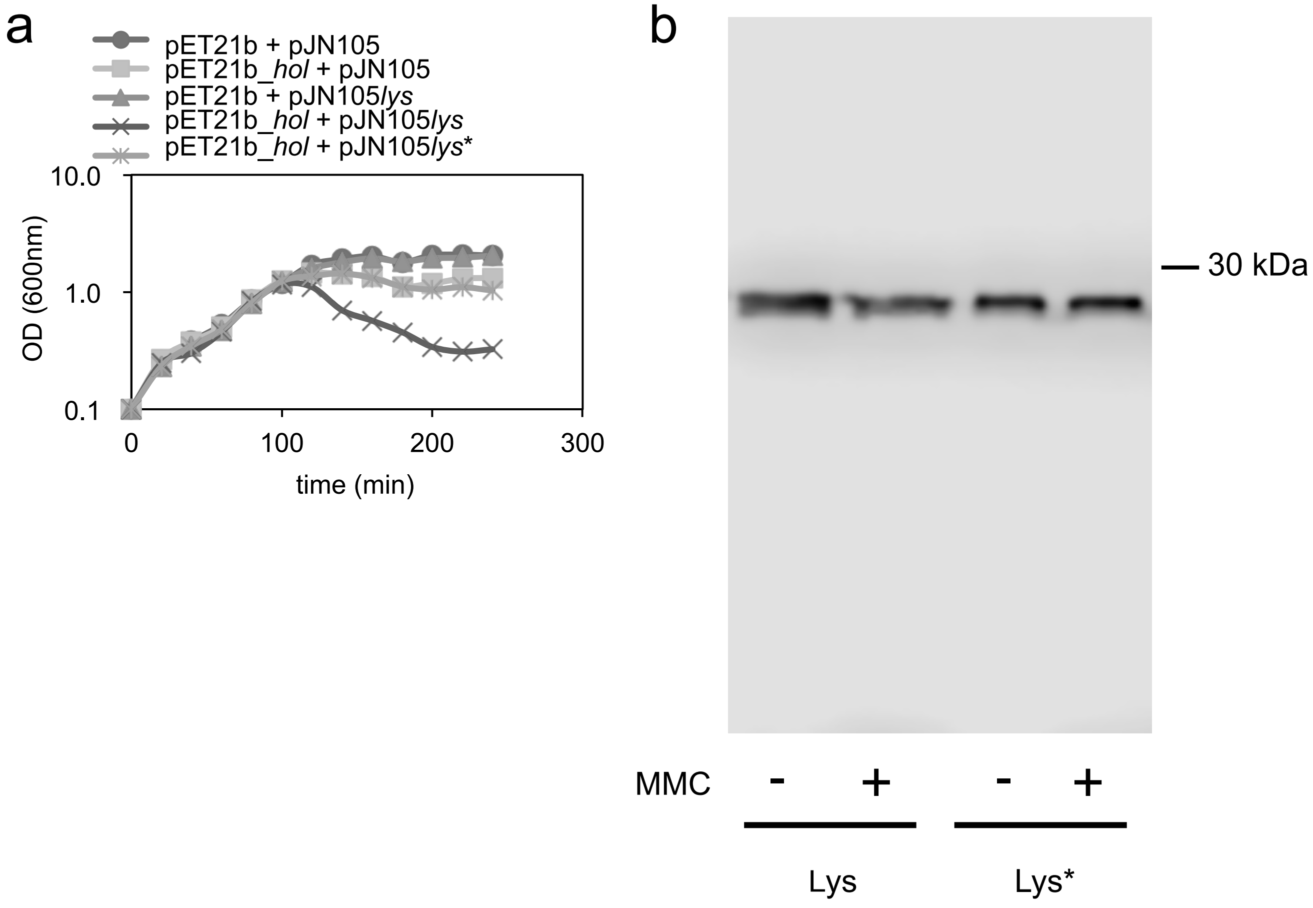

Supplementary Figure 2. Lys activity is required for cell lysis (a) Hol, Lys and Lys* were separately or jointly expressed in *E. coli* BL21(DE3). Cell lysis was induced by expressing both Hol (holin) and Lys (endolysin). Lys*, which carries a point mutation in the active site of the enzyme, did not induce cell lysis. Following 1h of incubation, expression of genes was induced by the addition of 0.1mM IPTG and 0.5% arabinose. Representative data of two independent experiments are shown. (b) Lys and Lys* expression levels are indistinguishable. Lys and Lys* were His tagged and their expression levels in the PAO1Δ*lys* mutant background were determined by Western blotting using anti-His antibodies. Samples were taken at the time point when MVs were collected. 2.5 µg of protein was used for SDS-PAGE.

a

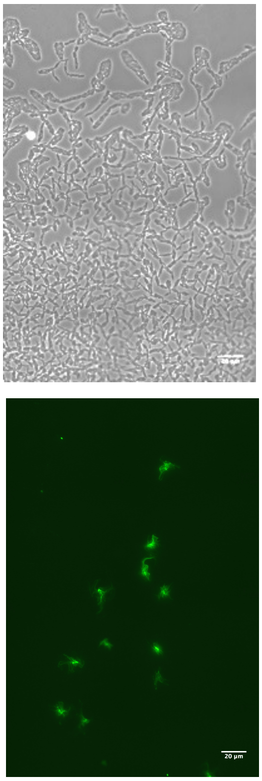

b

PA14 Gene	PAO1 Ortholog	Description
PA14_07980	PA0613	conserved hypothetical protein
PA14_08010	PA0616	putative baseplate assembly protein V
PA14_08030	PA0630	hypothetical protein
PA14_08040	PA0619	putative phage tail protein
PA14_08050	PA0620	putative tail fiber protein
PA14_08060	PA0621	putative tail length determinator protein
PA14_08070	PA0622	putative tail fiber assembly protein
PA14_08090	PA0623	putative phage tail tube protein
PA14_08100	PA0640	putative phage tail assembly protein
PA14_08120	PA0636	putative tail length determination protein
PA14_08130	PA0618	putative phage baseplate assembly protein
PA14_08180	PA0615	conserved hypothetical protein
PA14_08210	PA0633	putative major tail protein V
PA14_08220	PA0634	hypothetical protein
PA14_08230	PA0626	putative tail formation protein
PA14_08240	PA0647	conserved hypothetical protein
PA14_08260	PA0638	putative minor tail protein L
PA14_08270	PA0624	conserved hypothetical protein
PA14_08280	PA0635	hypothetical protein
PA14_08300	PA0641	putative phage-related protein, tail component
PA14_08330	PA0648	hypothetical protein
PA14_08320	PA0639	conserved hypothetical protein

Supplementary Figure 3. Pyocin structural genes are not required for eDNA release in interstitial biofilms. (a) Phase-contrast (top) and TOTO-1 stained eDNA (green, bottom) of interstitial biofilms of *P. aeruginosa* strain PA14. (b) Table showing PA14 mutants of pyocin structural genes examined for defects in eDNA release in interstitial biofilms. No defects were identified in any of the mutants indicating that the production of pyocins *per se* is not required for eDNA release through explosive cell lysis.

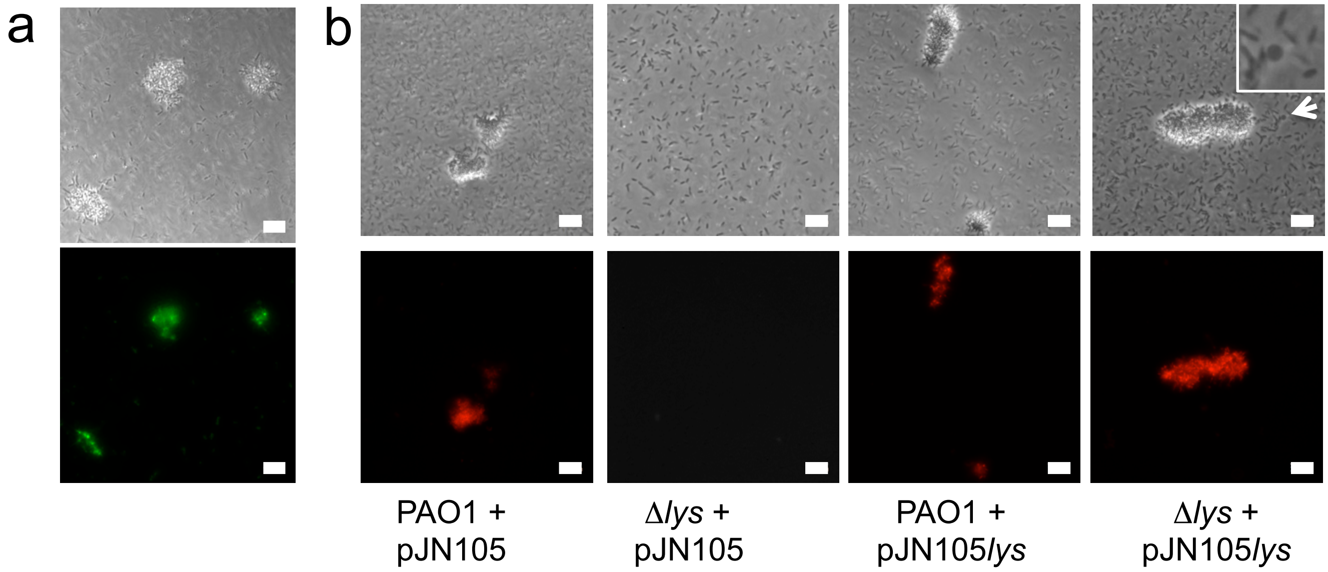

Supplementary Figure 4. Lys is required for microcolony formation in submerged biofilms. (a) Microcolonies in 8 h submerged biofilms of *P. aeruginosa* strain PAO1. Representative phase contrast (top) and eDNA (TOTO-1, bottom) images, scale bar 10 μm. (b) Microcolonies in 8 h submerged biofilms of PAO1 and PAO1Δ*lys* containing vector control (pJN105) or complementation plasmid (pJN105*lys*). Representative phase contrast (top) and eDNA (EthHD-2, bottom) images, scale bar 10 μm. Inset shows magnified view of round cell at arrow-head.

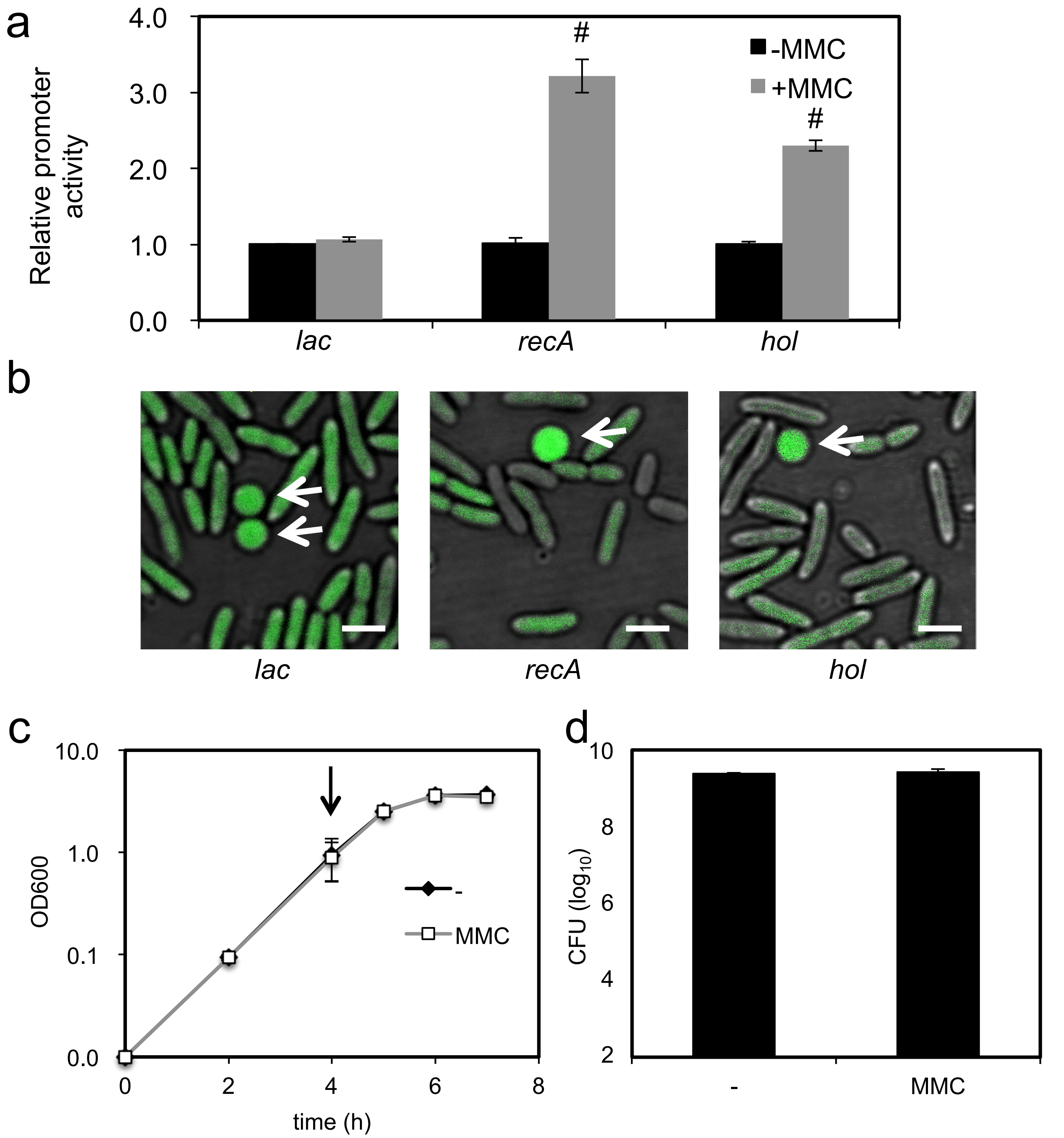

Supplementary Figure 5. Induction of *recA* and *hol* expression by MMC. (a) Promoter activities of transcriptional fusions to *eGFP* present on plasmids pMLAC-G, pMRECA-G and pM0614-G in *P. aeruginosa* PAO1 cultures were determined. Data represent relative eGFP fluorescence of cultures treated with 200 ng mL^{-1} MMC normalized against non-treated cultures. Values indicate the mean ± s.d. of three replicates. # $P < 0.0005$ versus MMC non-treated cultures (Student's *t* test). (b) *lacZ*, *recA* and *hol* promoter expression in induced cells. Arrows indicate rounded cells that express *eGFP*. Exponentially grown cells were treated for 1.5 h with MMC (200 ng mL^{-1}) in liquid culture, and further incubated for 1.5 h on a 0.5% agarose pad supplemented with LB and MMC (200 ng mL^{-1}); scale bar, 2 μm. (c) and (d) Bacterial growth was unaffected when cells were grown in the presence of 200 ng mL^{-1}MMC. (c) Growth curves indicating the time points when MMC (200 ng mL^{-1}) was added (arrow). (d) CFUs of the cultures after 7h of growth in absence or presence of MMC (200 ng mL^{-1}). MMC was added at the same time point as (c). Values indicate the mean ± s.d. of three replicates.

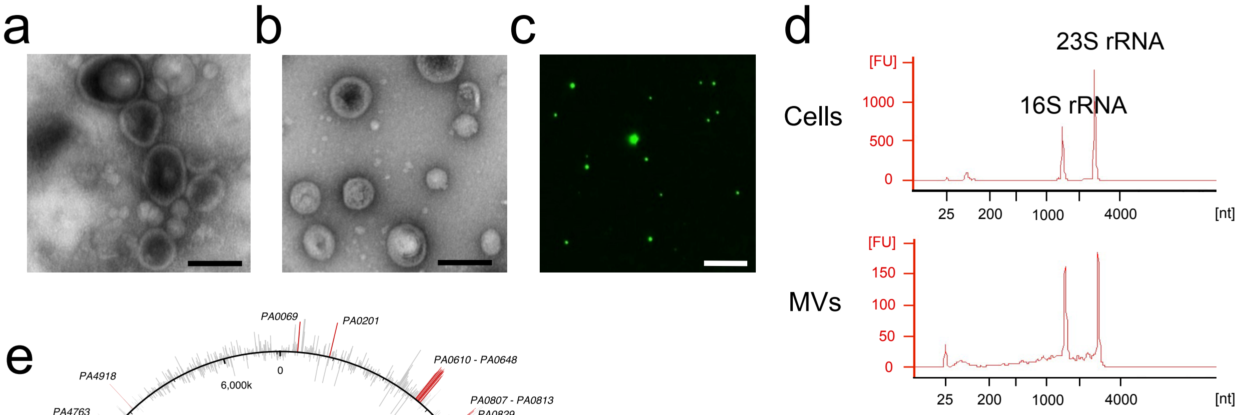

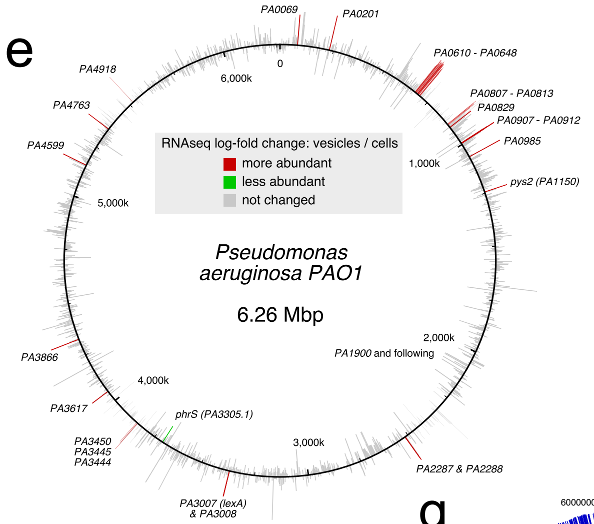

f

Gene	MVs vs cells (RNA)	MVs vs cells (DNA)
lexA	54±18	1.1
prtN	272±174	2
pyoS5	1017±151	-1.1
PA0617	2095±636	1.8
pys2	160±11	1.3
lys	445±106	1.3

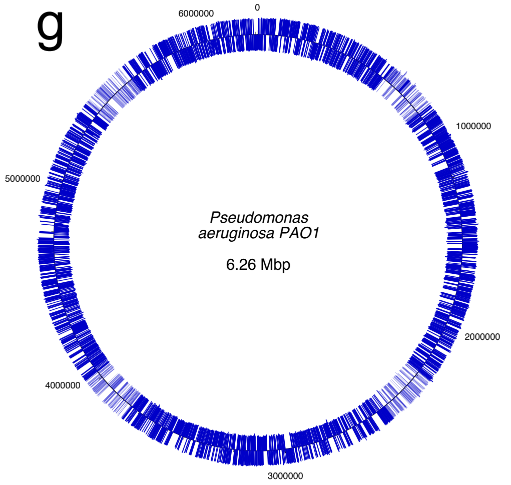

Supplementary Figure 6. DNA and RNA associated with MVs derived from *P. aeruginosa*. Transmission electron micrograph (TEM) of MVs collected from a stationary phase (a) and a MMC (b) treated *P. aeruginosa* PAO1 culture, scale bar, 100 nm. (c) Epifluorescence micrograph of purified MVs stained with SYTO RNASelect Green, scale bar, 10 µm. (d) The 23S and 16S rRNAs associated with MVs appear to be intact. The quality of the RNA collected from planktonic cells and purified MVs were analyzed with Agilent BioAnalyzer. The large peaks correspond to 16S rRNA and 23S rRNA. (e) Distribution of RNA-Seq reads of transcripts associated with MVs compared with transcripts of planktonic cells. The log-fold-changes of reads per transcript are plotted with respect to genomic location, with transcripts enriched in MVs pointing outwards and transcripts enriched in planktonic cells pointing inwards. More abundant transcripts associated with MVs are indicated in red and the less abundant transcript (*phrS*) is indicated in green. Differential abundance was determined as described in the Methods ("Illumina sequencing of RNA and DNA extracted from MVs"). (f) Table showing validation of the RNA-Seq data by qPCR of RNA isolated from purified MVs. Several transcripts that showed increased abundance in MVs relative to planktonic cells in the RNA-sequencing analysis were selected and their expression levels were validated by qPCR. The *rpoD* transcript was used for data normalization. The corresponding DNA was also analyzed as a control. Values indicate the mean fold-change ± s.d. of three replicates. (g) Distribution of DNA-seq reads of DNA associated with purified MVs. Log(RPKM) values are plotted with respect to genomic location, with the + strand on the outside and the - strand on the inside.

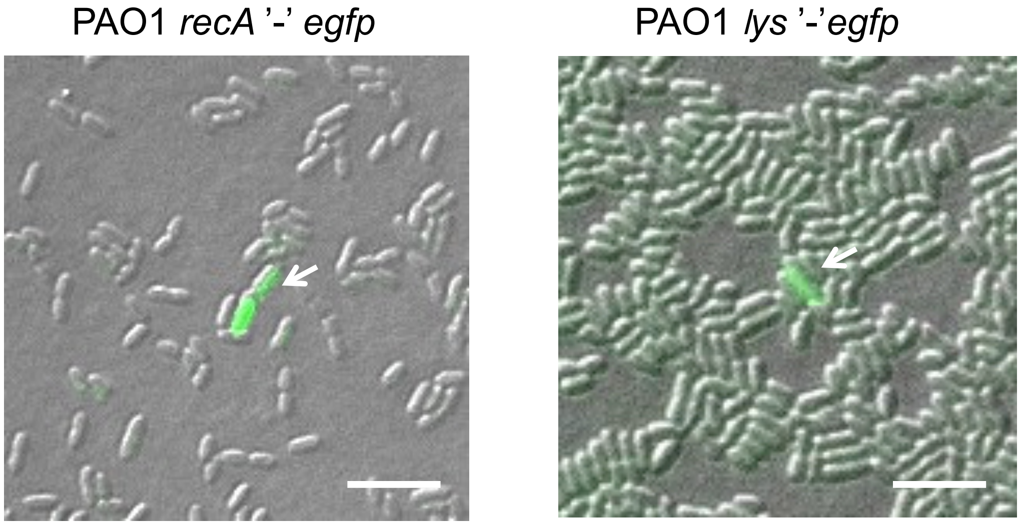

Supplementary Figure 7. Heterogeneous expression of SOS-regulated genes in a transcriptional single copy *eGFP* fusion strain. *eGFP* was transcriptionally fused to the 3′-end of *recA* and *lys* and integrated as single copy in the genome of *P. aeruginosa* PAO1. Planktonic cells were cultured under non-inducing conditions and examined with fluorescence microscopy. Arrow indicates cells with high levels of GFP expression showing heterogenous expression of *recA* and *lys*. These singly copy transcriptional fusions confirm the results obtained with the plasmid-based reporters shown in Fig. 7. However, the plasmid-born fusions gave rise to a much stronger fluorescent signal than the respective chromosomal fusions, scale bar, 5µm.

Supplementary Table 1. Strains and plasmids used in this study.

Strain, plasmid	Relevant characteristics	Source or reference
**Strains**		
E. coli		
K12 BW25113	*lacI*^{q}, rrnB$_{T14}$, Δ*lacZ*$_{WJ16}$, *hsdR*514, Δ*araBAD*$_{AH33}$, Δ*rhaBAD*$_{LD78}$	Keio Library
DH5α	*E. coli* strain for transformation (F^{-}, *lacZ*ΔM1, *recA1*)	TaKaRa
S17-1	Mobilizer strain	1
BL21(DE3)	F^{-}, *ompT*, *hsdS*$_{B}$ (r$_{B}^{-}$m$_{B}^{-}$), *gal*, *dcm*, (DE3)	TaKaRa
P. aeruginosa		
PAK	Wildtype	John Mattick, University of Queensland
PA14	Wildtype	Frederick Ausubel, Harvard University
PAO1	Wildtype also referred to as MPAO1	Colin Manoil, University of Washington
PAO1_Nott	Wildtype isogenic parent strain of PAO1*pqsA*	Paul Williams, University of Nottingham
PA103	Wildtype	ATCC
ATTC27853	Wildtype	ATCC
CF57	CF isolate	David Armstrong, Monash Children's Hospital
CF219	CF isolate	David Armstrong, Monash Children's Hospital
CF227	CF isolate	David Armstrong, Monash Children's Hospital
CF497	CF isolate	David Armstrong, Monash Children's Hospital
CF581	CF isolate	David Armstrong, Monash Children's Hospital
CLIN66	Endotracheal aspirate	Peter Midolo, Monash Medical Centre
CLIN67	Chest fluid	Peter Midolo, Monash Medical Centre
PAO1Δ*recA*	*recA* deletion mutant of PAO1	This study
PAO1Δ*lys*	PA0629 deletion mutant of PAO1	This study
PAKΔ*lys*	PA0629 deletion mutant of PAK	This study
PAO1ΔPA0620	PA0620 deletion mutant of PAO1	This study
PAO1ΔPA0622	PA0622 deletion mutant of PAO1	This study
PAO1ΔPA0641	PA0641 deletion mutant of PAO1	This study
PAO1::*recA*'-'*eGFP*	Genomic transcriptional fusion of *eGFP* to *recA*	This study
PAO1::*lys*'-'*eGFP*	Genomic transcriptional fusion of *eGFP* to PA0629	This study
PAO1*pqsA*	*pqsA* deletion mutant of PAO1_Nott	2
PA14_51430	PA14_51430::MAR2xT7; *pqsA*	3
PA14_08040	PA14_08040::MAR2xT7; putative phage tail protein	3
PA14_08050	PA14_08050::MAR2xT7; putative tail fiber protein	3
PA14_08300	PA14_08300::MAR2xT7; putative phage-related protein, tail component	3
PA14_08090	PA14_08090::MAR2xT7; putative phage tail tube protein	3
PA14_08210	PA14_08210::MAR2xT7; putative major tail protein V	3
PA14_08260	PA14_08260::MAR2xT7; putative minor tail protein L	3
PA14_08220	PA14_08220::MAR2xT7; hypothetical protein	3
PA14_08010	PA14_08010::MAR2xT7; putative baseplate assembly protein V	3
PA14_08330	PA14_08330::MAR2xT7; hypothetical protein	3
PA14_07980	PA14_07980::MAR2xT7; conserved hypothetical protein	3
PA14_08180	PA14_08180::MAR2xT7; conserved hypothetical protein	3
PA14_08030	PA14_08030::MAR2xT7; hypothetical protein	3
PA14_08130	PA14_08130::MAR2xT7; putative phage baseplate assembly protein	3
PA14_08230	PA14_08230::MAR2xT7; putative tail formation protein	3
PA14_08280	PA14_08280::MAR2xT7; hypothetical protein	3
PA14_08100	PA14_08100::MAR2xT7; putative phage tail assembly protein	3
PA14_08270	c PA14_08270::MAR2xT7; conserved hypothetical protein	3
PA14_08320	PA14_0832::MAR2xT7; conserved hypothetical protein	3
PA14_08240	PA14_08240::MAR2xT7; conserved hypothetical protein	3
PA14_08120	PA14_08120::MAR2xT7; putative tail length determinator protein	3
PA14_08060	PA14_08060::MAR2xT7; putative tail length determinator protein	3
PA14_08070	PA14_08070::MAR2xT7; putative tail fiber assembly protein	3
**Plasmids**		
pUCPSK	*E. coli-Pseudomonas* shuttle vector, Ap^{R}	4
pUCPKS	*E. coli-Pseudomonas* shuttle vector, Ap^{R}	4
pmCherry-C2	Source of mCherry fluorescent protein gene (*mCHFP*)	CLONTECH
pUCPCFP	*ecfp* sub-cloned into pUCPSK	5
pUCPmChFP	*mChFP* from pmCherry-C2 sub-cloned into pUCPKS	This study
pUC19	Cloning vector, Ap^{r}	TakaRa
pJN105	Broad host range arabinose inducible gene expression vector	6
pET21b	Expression vector, Ap^{r}	Novagen
pJN105*lys*	Arabinose inducible Lys-His expression vector	This study
pJN105*lys**	Arabinose inducible Lys*-His expression vector, Lys catalytic site mutated	This study
pET21b_*hol*	Hol-His expression vector	This study
pG19II	pK19mobsac derived suicide vector; *sacB* Gm^{r}	7
pG19recA	*recA* deletion cassette in pG19II	8
pG19PA0629	PA0629 deletion cassette in PG19II	This study
pG19PA0620	PA0620 deletion cassette in PG19II	This study
pG19PA0622	PA0622 deletion cassette in PG19II	This study
pG19PA0641	PA0641 deletion cassette in PG19II	This study
pG19_*recA*'-'eGFP	*recA'-'eGFP* transcriptional fusion cassette in pG19II	This study
pG19_*lys*'-'eGFP	PA0629*'-'eGFP* transcriptional fusion cassette in pG19II	This study
pEGFP	Plasmid harboring *eGFP*	CLONTECH
pMEXGFP	pMEX9 derived promoter-probe vector; *eGFP,* Gm^{r}	8
pMLAC-G	*lac* promoter region fused to *eGFP* in pMEXGFP	This study
pMRECA-G	*recA* promoter region fused to *eGFP* in pMEXGFP	This study
pM0614-G	*hol* promoter region fused to *eGFP* in pMEXGFP	8

Supplementary Table 2. Primers used in this study.

Primers	Sequence 5 '→ 3' (restriction enzyme sites are underlined)
**Gene deletion**	
ΔPA0620_F1	CCTAAGCTTGGGCTGCGAAGTGCTGATCAGCGTGCTCG
ΔPA0620_R1	CGGGATCCGGGCAGGCCACCGTATTTCGGAGTATTGGTCG
ΔPA0620_F2	CGGGATCCGAGGTGATTCGCAATGGCTACTTTGCTCAGGC
ΔPA0620_R2	GCTCTAGAGGCTGGGCATTGAAGCGACTCTTGCCGTCG
ΔPA0622_F1	CCTAAGCTTGGGCCACCATCCGCACATAACCATGACGTCG
ΔPA0622_R1	CGGGATCCGTAGGTAGATCTCCATTAATGAAAAACCCCGCACG
ΔPA0622_F2	CGGGATCCGGCAGTGGCTCACCGAAGTTCTGGATGTCGC
ΔPA0622_R2	GCTCTAGAGGCGGTTCGTCTCGTCTGTTATCGATGTCCG
ΔPA0629_F1	CCCAAGCTTGGTGCAACCGAGTTTCCGTATCGTTGCCGACG
ΔPA0629_R1	CGGGATCCGGCGATCCTCCTGCACTCCGATGGGTTTCAG
ΔPA0629_F2	CGGGATCCTGCGGTCACGGGCTCAACGAGCTGGC
ΔPA0629_R2	GCTCTAGAGTGTTGAACGAGGTCACGCCTTCGATCTCCAG
ΔPA0641_F1	GCAACTGCAGCTGGCATCTACCTGGGCAATGACTGGCG
ΔPA0641_R1	GCTCTAGAGGTCGTGATGGTCTTGTTCATGACGTTCCTTCAGGC
ΔPA0641_F2	GCTCTAGAGGAGCAGGCCAGGTCAGAGTTGAGATGGG
ΔPA0641_R2	CGGGATCCGGCGCTTCGGAATTCAGCGTGACGGAAACCG
***eGFP* genomic fusion**	
recAG_F1	GGAATTCCGTCGAGATCTACGGTCCGGAATCC
recAG_R1	CGCCTCGAGTCAATCGGCTTCGGCGTCAGCC
recAG_F2	GACTAGTGGAGGCCAATGGCGATCGTGCTCGATAC
recAG_R2	GCTCTAGACGCCCAGTTCTACCGCAACTTCCAC
0629G_F1	GGAATTCAGCCTGCTGCTCCAAGGCTTCAAGG
0629G_R1	CGCCTCGAGTCATACAGCACCGCCCTGGCC
0629G_F2	GACTAGTGGAGGTTCCATGAGCCGGCTCGCTCTGCTCC
0629G_R2	GCTCTAGACCCGAGCCGTTGTCGGATGCAAAC
egfp_F2	CGCCTCGAGGGAGGTCCATGGTGAGCAAGGGCGAGGAGCTGTTC
egfp_R2	GACTAGTTTACTTGTACAGCTCGTCCATGCCGAGAG
**Promoter assay**	
pLac_F	CGGAATTCTGTTCTTTCCTGCGTTATCC
pLac_R	CGGGATCCGCTGTTTCCTGTGTGAAATTG
pRecA_F	CGGGATCCGGAAGTGGTCGAGGCCATGGTGC
pRecA_R	CGCAAGCTTCGAGGATTCCGGACCGTAGATCTCG
**Expression plasmids**	
PA0629AA_F	GAGGTCAAGAGCGTCGAGTTGAAGAACC
PA0629_E51V_F	GTCGGCCACGTAAGCAGCCAGTTGAC
PA0629-E51V_R	CCGGGTCAACTGGCTGCTTACGTG
cPA0629H_F	CTGAATTCCTGAAACCCATCGGAGTGCAGGAGGATCG
cPA0629H_R	CCTCTAGATCAGTGGTGGTGGTGGTGGTGTGACAGCACCGCCCTGGC
PA0614b_F	GATTCGACATATGAAGCACCGGAACCCGGCCCTG
PA0614b_R	CCGCTCGAGATGCGGGCCACGGTCGTCCG
mCherry_F	ATAGCATGCTGAGCAAGGGCGAGG
mCherry_R	CGCAAGCTTACTTGTACAGCTCGTCC
**qPCR primers**	
prtN (PA0610)_F	ATTGGTCTACCGCATCTTCG
prtN (PA0610)_R	TGCATGGCCTTGTGACTATC
lexA (PA3007)_F	AAGCCGAGATCCTCTCCTTC
lexA (PA3007)_R	CCGGAGTCATTTCGATGG
pyoS5 (PA0985)_F	AACTGGAGCGGGACTACAGA
pyoS5 (PA0985)_R	TGCTTGCGTAACCAGTCTTG
PA0617_F	GGCCTGGCTCATCTTAAACA
PA0617_R	TTTCCAGCCTTCGCTCAC
pys2 (PA1150)_F	ACGGCTTCAGACTTTCCTCA
pys2 (PA1150)_R	AGATAGCGATTTGCGCCTTA
PA0614_F	CGCTCTGGGTACTGATCCTG
PA0614_R	CGTTCGTAGAGACCGACTGC
PA0629_F	GACGAGAGGGAGATCGACAC
PA0629_R	GTAGGTGTTGTCGGCAATCG
PA0576_F	CATCGCCAAGAAGTACACCA
PA0576_R	GCGACGGTATTCGAACTTGT

Supplementary References

1 Simon, R., O'Connell, M., Labes, M. & Pühler, A. Plasmid vector for the genetic analysis and manipulation of rhizobia and other gram-negative bacteria. *Methods Enzymol* **118**, 640-659 (1986).

2 Aendekerk, S. *et al.* The MexGHI-OpmD multidrug efflux pump controls growth, antibiotic susceptibility and virulence in *Pseudomonas aeruginosa* via 4-quinolone-dependent cell-to-cell communication. *Microbiology* **151**, 1113-1125 (2005).

3 Liberati, N. T. *et al.* An ordered, nonredundant library of *Pseudomonas aeruginosa* strain PA14 transposon insertion mutants. *Proc Natl Acad Sci USA* **103**, 2833-2838 (2006).

4 Watson, A. A., Alm, R. A. & Mattick, J. S. Construction of improved vectors for protein production in *Pseudomonas aeruginosa*. *Gene* **172**, 163-164 (1996).

5 Gloag, E. S. *et al.* Self-organization of bacterial biofilms is facilitated by extracellular DNA. *Proc Natl Acad Sci USA* **110**, 11541-11546 (2013).

6 Newman, J. R. & Fuqua, C. Broad-host-range expression vectors that carry the L-arabinose-inducible *Escherichia coli araBAD* promoter and the *araC* regulator. *Gene* **227**, 197-203 (1999).

7 Maseda, H. *et al.* Enhancement of the *mexAB-oprM* efflux pump expression by a quorum-sensing autoinducer and its cancellation by a regulator, MexT, of the *mexEF-oprN* efflux pump operon in *Pseudomonas aeruginosa*. *Antimicrob Agents Chemother* **48**, 1320-1328 (2004).

8 Toyofuku, M. *et al.* Membrane vesicle formation is associated with pyocin production under denitrifying conditions in *Pseudomonas aeruginosa* PAO1. *Environ Microb* **16**, 2927-2938 (2014).
